# Mucoid Pseudomonas aeruginosa Can Produce Calcium-Gelled Biofilms Independent of the Matrix Components Psl and CdrA

**DOI:** 10.1128/jb.00568-21

**Published:** 2022-04-13

**Authors:** Holly M. Jacobs, Lindsey O’Neal, Edward Lopatto, Daniel J. Wozniak, Thomas Bjarnsholt, Matthew R. Parsek

**Affiliations:** a Molecular and Cellular Biology Graduate Program, University of Washington, Seattle, Washington, USA; b Department of Microbiology, University of Washington, Seattle, Washington, USA; c Department of Microbial Infection and Immunity, The Ohio State University, Columbus, Ohio, USA; d Department of Microbiology, The Ohio State University, Columbus, Ohio, USA; e Costerton Biofilm Center, Department of Immunology and Microbiology, Faculty of Health and Medical Science, University of Copenhagen, Copenhagen, Denmark; f Department of Clinical Microbiology, Rigshospitalet, Copenhagen, Denmark; University of California—San Francisco

**Keywords:** *Pseudomonas aeruginosa*, biofilms, alginate, calcium, Psl, CdrA, c-di-GMP, cystic fibrosis, mucoid

## Abstract

Biofilms are aggregates of microorganisms embedded in an extracellular matrix comprised largely of exopolysaccharides (EPSs), nucleic acids, and proteins. Pseudomonas aeruginosa is an opportunistic human pathogen that is also a model organism for studying biofilms in the laboratory. Here, we define a novel program of biofilm development used by mucoid (alginate-overproducing) P. aeruginosa in the presence of elevated calcium. Calcium cations cross-link negatively charged alginate polymers, resulting in individual cells being suspended in an alginate gel. The formation of this type of structurally distinct biofilm is not reliant on the canonical biofilm EPS components Psl and Pel or the matrix protein CdrA. We also observed that mucoid P. aeruginosa biofilm cells do not have the typical elevated levels of the secondary messenger cyclic di-GMP (c-di-GMP), as expected of biofilm cells, nor does the overproduction of alginate rely on high c-di-GMP. This contrasts with nonmucoid biofilms in which the production of the matrix components Psl, Pel, and CdrA is positively regulated by elevated c-di-GMP. We further demonstrate that calcium-gelled alginate biofilms impede the penetration of the antibiotic tobramycin, thus protecting the biofilm community from antibiotic-mediated killing. Finally, we show that bacterial aggregates with a dispersed cell arrangement like laboratory-grown calcium-alginate biofilm structures are present in explanted cystic fibrosis (CF) lung samples. Our findings illustrate the diverse nature of biofilm formation and structure in P. aeruginosa.

**IMPORTANCE** The opportunistic pathogen Pseudomonas aeruginosa produces a complex biofilm matrix comprised of exopolysaccharides (EPSs), nucleic acids, and proteins. P. aeruginosa biofilm formation canonically depends on a variable combination of the exopolysaccharides Psl and Pel and the matrix protein CdrA. We demonstrate that mucoid P. aeruginosa, which overproduces the EPS alginate, possesses an entirely alternate and calcium-dependent method of biofilm formation. These mucoid biofilm structures do not require Psl, Pel, or CdrA, and they display a unique organization of individually suspended cells similar to bacterial aggregates observed in cystic fibrosis airways. Furthermore, calcium-gelled mucoid biofilms impede the penetration and killing action of the antibiotic tobramycin, illustrating their potential clinical significance. Our findings highlight the compositional and structural variety of P. aeruginosa biofilm aggregates.

## INTRODUCTION

Individuals with cystic fibrosis (CF) are subject to multiple deleterious effects due to a defective chloride ion channel, including a buildup of mucus in their airways, which leads to chronic bacterial infections (1, 2). These infections are linked to biofilms produced by bacterial pathogens such as Pseudomonas aeruginosa and Staphylococcus aureus (2, 3). The biofilm lifestyle provides advantages such as tolerance to antibiotics and environmental stressors as well as protection from the host immune system (4). The P. aeruginosa biofilm matrix is a complex, multicomponent mesh encasing cells in a variety of exopolysaccharides (EPSs) (Psl, Pel, and alginate) in addition to matrix proteins (CdrA, LecB, and ecotin), extracellular DNA (eDNA), and outer membrane vesicles (5–8). The production of Psl, Pel, and CdrA are positively controlled by the secondary messenger bis-(3′-5′)-cyclic dimeric GMP (c-di-GMP) (9). In P. aeruginosa, the current paradigm is that high levels of intracellular c-di-GMP are associated with biofilm matrix production (particularly EPS and CdrA) and aggregate formation.

The EPS Psl, a neutral, mannose-rich polysaccharide, is important for the initial attachment of cells to a surface and each other. Psl localizes primarily to the aggregate periphery where it interacts with the matrix protein CdrA (10). CdrA serves important roles in biofilm integrity and stabilization through its interactions with Psl and Pel (11, 12). Pel, a positively charged polysaccharide of *N-*acetylglucosamine and *N*-acetylgalactosamine, is capable of interacting with eDNA (13). These findings have been carefully described in clinical and environmental nonmucoid strains (14).

Alginate-overproducing (mucoid) P. aeruginosa strains have a slimy, mucous-like appearance when grown on solid medium and commonly arise over time in chronically infected individuals with CF (15, 16). The emergence of these strains is correlated with a worse long-term prognosis and is often associated with advanced stages of disease (17). Additionally, mucoid P. aeruginosa can have a higher tolerance to antibiotics and may facilitate cocolonization with other common CF-associated microbes (18–20). Alginate is a negatively charged polymer of variably acetylated mannuronic acid (M) and guluronic acid (G) arranged in poly(M) and poly(MG) blocks (21). Alginate production is positively controlled by the alternative sigma factor AlgU, which in nonmucoid strains is inactivated through sequestration by the anti-sigma factor MucA (22). Mutations in *mucA* can result in the release of AlgU and the subsequent regulation of the sizable AlgU regulon (23, 24). The biofilm matrix of mucoid strains is poorly understood relative to that of nonmucoid strains, although Psl is thought to play a prominent role (25).

As in eukaryotes, calcium is an important signal to prokaryotes, although its precise roles remain understudied in most species (26, 27). Intracellular levels of calcium are tightly controlled in P. aeruginosa, and growth in elevated calcium may enhance mucoid biofilm formation and induces the production of certain virulence factors (28–30). Extracellular calcium can also cross-link P. aeruginosa alginate, resulting in the formation of a gelled matrix (31). P. aeruginosa alginate lacks poly(G) blocks, which facilitate the “eggbox” model of chelation seen in seaweed-derived alginates, so alginate gelation occurs by calcium cross-linking MG blocks between alginate polymers instead (21, 32). When mucoid P. aeruginosa strains are grown with CF airway-approximate levels of calcium (1 to 5 mM), alginate-calcium cross-linking can occur, resulting in a gelled biofilm matrix (28, 33, 34).

In this study, we examine mucoid biofilm formation and structure. First, we demonstrate that calcium-cross-linked mucoid P. aeruginosa biofilms are structurally unique, with individual cells being suspended within an alginate matrix. We then show that the critical nonmucoid biofilm matrix components CdrA, Psl, and Pel are not required for the formation of these calcium-gelled alginate biofilms. We next demonstrate that mucoid biofilms and alginate production do not require high c-di-GMP. Furthermore, calcium-gelled alginate colony biofilms protect cells against tobramycin killing and impede the penetration of the antibiotic more than nongelled mucoid biofilms or nonmucoid biofilms. Finally, we also show that mucus plugs from explanted CF lungs contain P. aeruginosa aggregates possessing a cell spacing and appearance similar to those of the calcium-gelled mucoid biofilms observed in the laboratory setting. Collectively, these data suggest that mucoid P. aeruginosa can produce biofilms with clinically relevant properties distinct from those of the biofilm aggregates produced by nonmucoid strains.

## RESULTS

### Mucoid P. aeruginosa strains use a distinct, calcium-dependent pattern of biofilm development.

**FIG 1:**
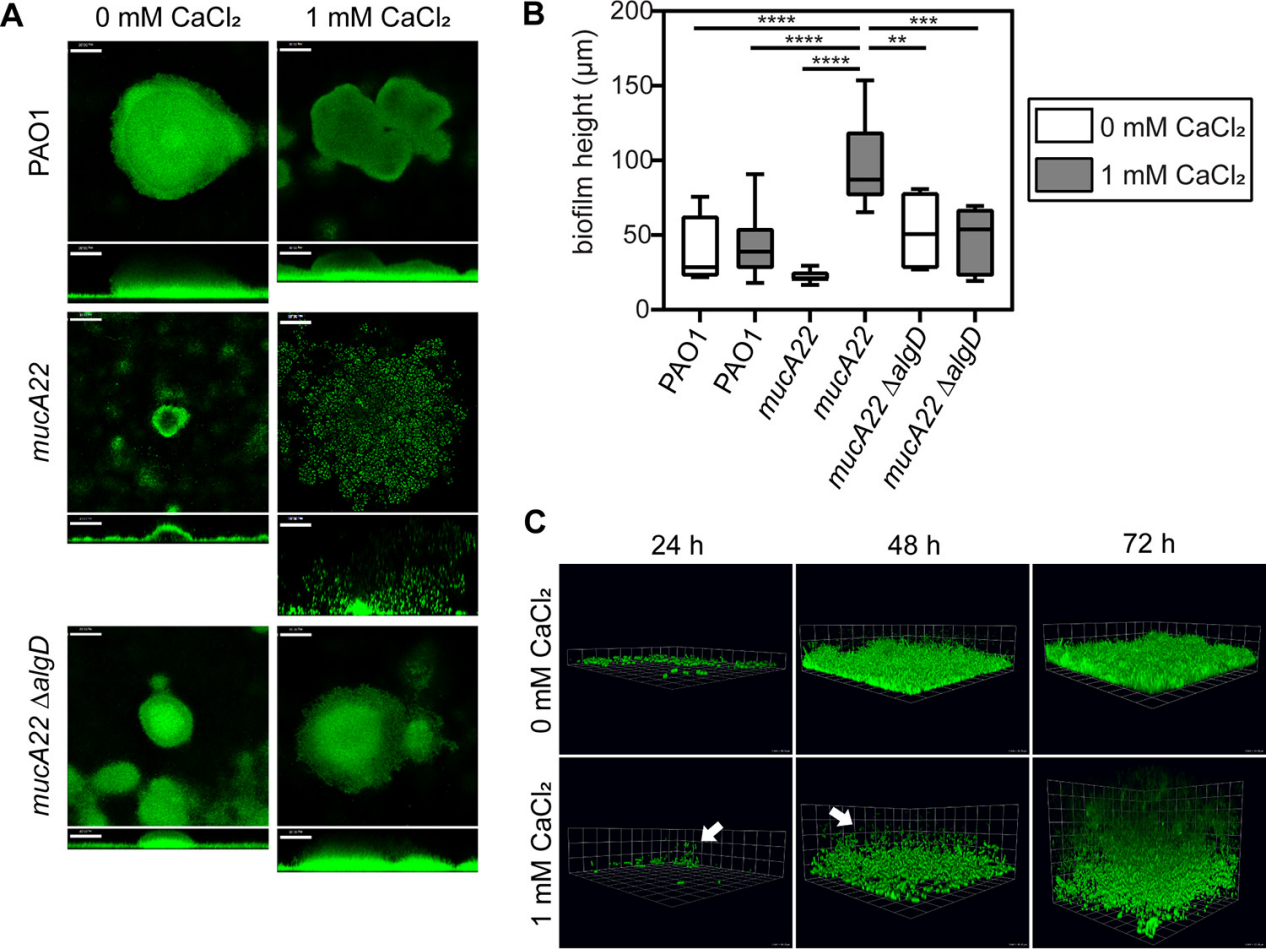
Calcium-cross-linked P. aeruginosa mucoid biofilms are structurally distinct. (A) Representative images from biofilms cultivated for 72 h under continuous-flow conditions. Cells are constitutively expressing GFP (pseudocolored green). Horizontal cross sections (squares) from the middle of the biomass and sagittal views (rectangles below the cross sections) are shown. Magnification, ×200. Bars, 50 μm. (B) Quantification of biofilm height (in micrometers) measured from the base of the biofilm to its highest point at 72 h. Quantified images are from three biological replicates with 3 to 4 images measured per replicate for each genotype and condition. White boxes indicate growth with 0 mM CaCl_2_, and gray boxes indicate growth with 1 mM CaCl_2_. Boxes extend from the 25th to the 75th percentiles, the whiskers represent the minimum and maximum values, and the line in the middle of the box is plotted at the median value. **, *P* < 0.01; ***, *P* < 0.001; ****, *P* < 0.0001 (by one-way ANOVA performed with multiple comparisons). (C) Three-dimensional renderings of representative confocal images from PAO1 *mucA22* biofilms cultivated under continuous-flow conditions and imaged at 24, 48, and 72 h postinoculation. White arrows indicate cells stably tethered above the surface of the coverslip at 24 h and 48 h of growth with calcium, presumably remaining associated through calcium-cross-linked alginate. Magnification, ×630. Grid square sides represent 10.16 μm.

The effect of calcium on mature, mucoid biofilm structure has been previously described (28, 35). However, mucoid biofilm characteristics at early stages following initial attachment were unknown. To investigate this, three isogenic P. aeruginosa strains, PAO1 (nonmucoid; AlgU is inactivated), PAO1 *mucA22* (mucoid; AlgU is active), and PAO1 *mucA22* Δ*algD* (nonmucoid; AlgU is active, but alginate cannot be produced), were grown under continuous-flow conditions. Mature 72-h-old biofilms from nonmucoid and mucoid strains are structurally distinct from each other (Fig. 1A). PAO1 and the *mucA22* Δ*algD* strain formed densely packed, surfaced-adhered aggregates regardless of calcium. As in previous studies, in the presence of calcium, the mucoid strain PAO1 *mucA22* produced gelled biofilm structures characterized by suspended, individual cells (28). This was distinct from PAO1 *mucA22* grown without calcium, which formed comparatively small aggregates that carpeted the coverslip surface. The average biofilm height emphasizes the thickness of calcium-gelled mucoid biofilms, which many times extended >100 μm into the lumen of the flow cell (Fig. 1B). In contrast, the mean height of nonmucoid biofilms at 72 h was 42 to 52 μm, and mucoid biofilms grown without calcium had aggregates averaging 22 μm (Fig. 1B). There were no significant growth differences for these strains in the presence or absence of 1 mM CaCl_2_ (see Fig. S1A and B in the supplemental material).

For many bacterial species, a consistent pattern of biofilm development is observed on abiotic surfaces. Commonly observed stages include initial attachment, transition to irreversible attachment, microaggregate or microcolony formation, and, ultimately, the formation of densely packed, large cellular aggregates (36). Our observations suggest that mucoid biofilm formation follows a different pattern in the presence of calcium. To investigate this, we monitored PAO1 *mucA22* biofilm development after initial attachment. In the absence of calcium, a monolayer of attached cells began to accumulate on the surface, with evidence of initial microcolony formation by 24 h. This is consistent with patterns of biofilm formation observed in nonmucoid strains (Fig. S2). However, when grown with calcium, mucoid strains did not form microcolonies at this time point. Instead, individual cells suspended above the surface could be observed at early stages (Fig. 1C, indicated by white arrows). These cells are presumed to be tethered by calcium-cross-linked polymers of alginate. The difference between PAO1 *mucA22* biofilms grown with and without calcium is abundantly clear by 48 and 72 h of growth (Fig. 1C; Fig. S2).

**FIG 2:**
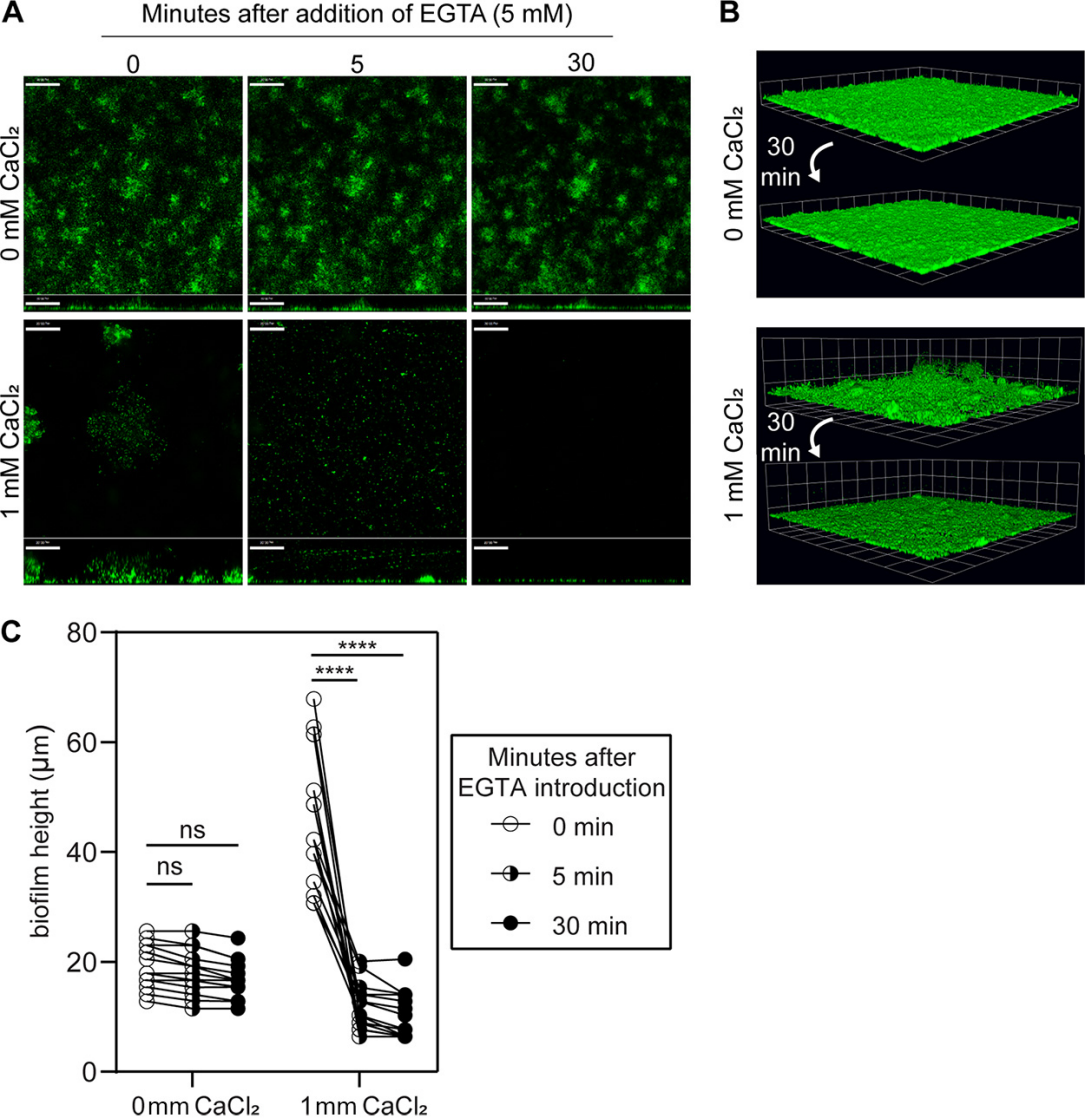
Calcium chelation rapidly disrupts calcium-cross-linked mucoid biofilms. (A) Representative confocal images from PAO1 *mucA22* biofilms cultivated for 48 h in flow cells throughout 30 min of EGTA treatment (5 mM). Images were captured 0, 5, and 30 min after the initial introduction of EGTA. Cells are constitutively expressing GFP (pseudocolored green). Horizontal cross sections (squares) from the middle of the biomass and sagittal views (rectangles) are shown from the same relative z-stack for each time point. Magnification, ×200. Bars, 50 μm. (B) Three-dimensional renderings of representative confocal images from 48-h-old PAO1 *mucA22* biofilms before and after 30 min of EGTA exposure. Magnification, ×200. Grid square sides represent 32.01 μm. (C) Quantification of biofilm height (in micrometers) measured from the base of the biofilm to its highest point 0, 5, and 30 min after EGTA treatment demonstrates that mucoid calcium-gelled biofilms lose much of their height after EGTA treatment. Images quantified were from three biological replicates with 1 to 2 biofilms from 3 to 4 images measured per condition per replicate. Each line represents a single aggregate measured at each time point. ****, *P* < 0.0001; ns, not significant (*P* > 0.05) (by an unpaired, two-tailed *t* test).

We hypothesized that much of the space between suspended cells in PAO1 *mucA22* biofilms grown with calcium is filled with calcium-cross-linked alginate. To test this, we treated 48-h-old calcium-gelled mucoid biofilms with the calcium chelator EGTA and assessed the effect on biofilm stability. We observed that the addition of EGTA quickly resulted in a pronounced loss of biofilm biomass (Fig. 2A to C). In contrast, the addition of EGTA to *mucA22* biofilms grown in the absence of elevated calcium had little impact on the microaggregate structures (Fig. 2A to C). Likewise, the addition of EGTA had a minimal effect on *mucA22* Δ*algD* 48-h-old biofilms regardless of their growth with excess calcium (Fig. S3A and B). We also tested the stability of *mucA22* and *mucA22* Δ*algD* biofilms grown with calcium by switching 48-h-old biofilms to growth medium lacking calcium. This switch from 1 to 0 mM CaCl_2_ had no effect on *mucA22* Δ*algD* biofilms, while the *mucA22* strain still experienced a loss of biomass although much slower than what was observed during EGTA treatment (Fig. S3C). These data suggest that mucoid P. aeruginosa biofilms are reliant on the continued presence of calcium to maintain their unique gelled structure.

### Key nonmucoid biofilm matrix components are not required for calcium-gelled alginate biofilms.

**FIG 3:**
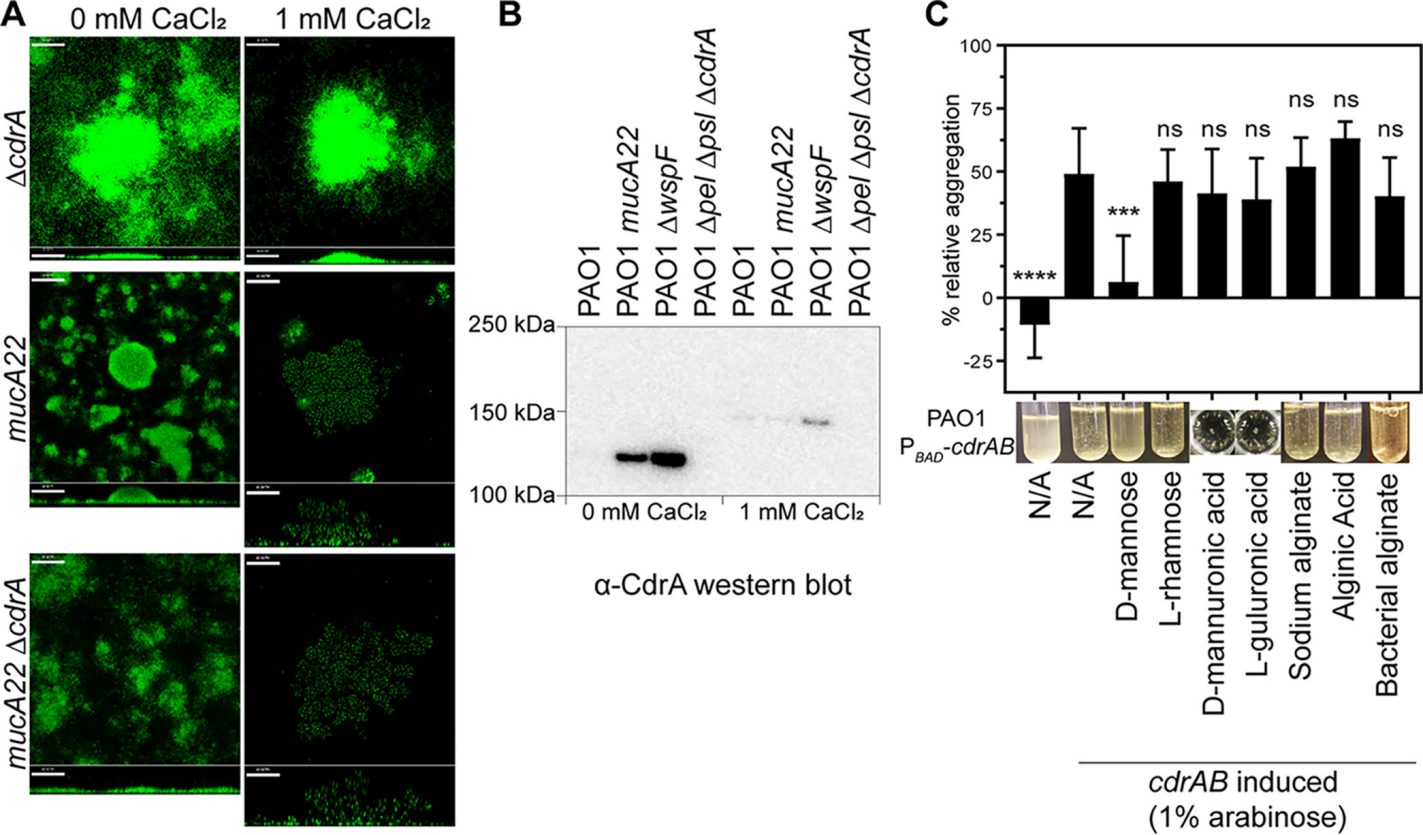
The matrix protein CdrA is not required for calcium-cross-linked mucoid biofilms. (A) Representative confocal images from biofilms cultivated under continuous-flow conditions and imaged 72 h after inoculation of the flow cell chamber in growth medium with or without additional calcium. Cells are constitutively expressing GFP (pseudocolored green). Horizontal cross sections (squares) from the middle of the biomass and sagittal views (rectangles) are shown. Magnification, ×200. Bars, 50 μm. (B) Anti-CdrA Western blotting of cultures grown statically in EPRI medium for 24 h at 37°C with or without added calcium showing that the abundance of released CdrA is greatly decreased upon the addition of calcium to the growth medium. A total of 0.6 μg of total protein was loaded for each sample. (C) Alginate does not interfere with CdrA-mediated aggregation. PAO1 P*_BAD_*-*cdrAB* was subcultured with the indicated sugar (bacterial alginate, 0.5 mg/mL; others, 5 mg/mL) and incubated with shaking at 37°C for 2 to 2.5 h. All cultures except for the leftmost (uninduced) culture also contained 1% arabinose. d-Mannose was included as a positive control (disrupts aggregation), and l-rhamnose was included as a negative control (does not disrupt aggregation). Aggregation was visually assessed (representative images of tubes or plate wells are shown below the bars) and then quantified by measuring the optical density at 600 nm (OD_600_). Percent relative aggregation was calculated using the following equation: % relative aggregation = (OD_vector control_ − OD_sample_)/OD_vector control_ × 100. One-way ANOVA was performed comparing the 1% arabinose conditions to the other tested conditions (ns, not significant [*P* > 0.05]; *, *P* < 0.05; ***, *P* < 0.001). N/A, not applicable.

Nonmucoid biofilm structures typically rely on the production of multiple matrix components, including the exopolysaccharides Psl and Pel and the protein CdrA. Studies have also suggested an important role for these matrix factors in mucoid biofilms (25), so we sought to investigate how these components contribute to the calcium-gelled alginate biofilm structure. We first examined the role of the biofilm matrix protein CdrA. Similar to the nonmucoid strain PAO1 Δ*cdrA*, when PAO1 *mucA22* Δ*cdrA* was grown without excess calcium, only a carpet of cells punctuated with small microaggregates was observed (Fig. 3A). However, in the presence of calcium, PAO1 *mucA22* Δ*cdrA* produced gelled biofilms that appeared structurally identical to those of PAO1 *mucA22* grown with 1 mM CaCl_2_.

To test whether CdrA is present in the biofilm matrix under these growth conditions, Western blotting was performed on statically grown biofilm cultures. In the absence of calcium, CdrA was abundant in PAO1 *mucA22* and PAO1 Δ*wspF* (a strain known to overproduce CdrA) biofilms but was present at very low levels when the same strains were grown with 1 mM CaCl_2_ (Fig. 3B). While released CdrA typically has a molecular weight of 150 kDa, due to extracellular proteolysis, it is processed to a smaller ∼120-kDa form (11). However, it appears that the presence of calcium reduces CdrA processing, resulting in the larger 150-kDa form (Fig. 3B).

Finally, we investigated whether CdrA directly interacts with alginate. Previous work has shown that CdrA directly binds to the EPS components Psl and Pel (10, 12). Experimentally, this binding can be interrogated by exploiting the fact that CdrA overexpression results in cellular aggregation via CdrA-CdrA interactions. CdrA-CdrA interactions can be disrupted by adding either purified EPS or the monosaccharides found in that EPS (10). Therefore, we used a strain containing arabinose-inducible CdrA and attempted to disrupt CdrA-mediated aggregation by adding alginate or its individual sugars, d-mannuronic acid and l-guluronic acid. However, neither the addition of alginate nor its monosaccharides interfered with CdrA-mediated aggregation (Fig. 3C). Together, these data indicate that CdrA does not directly interact with alginate, nor is it a crucial component of calcium-cross-linked alginate gels formed by the mucoid strain.

**FIG 4:**
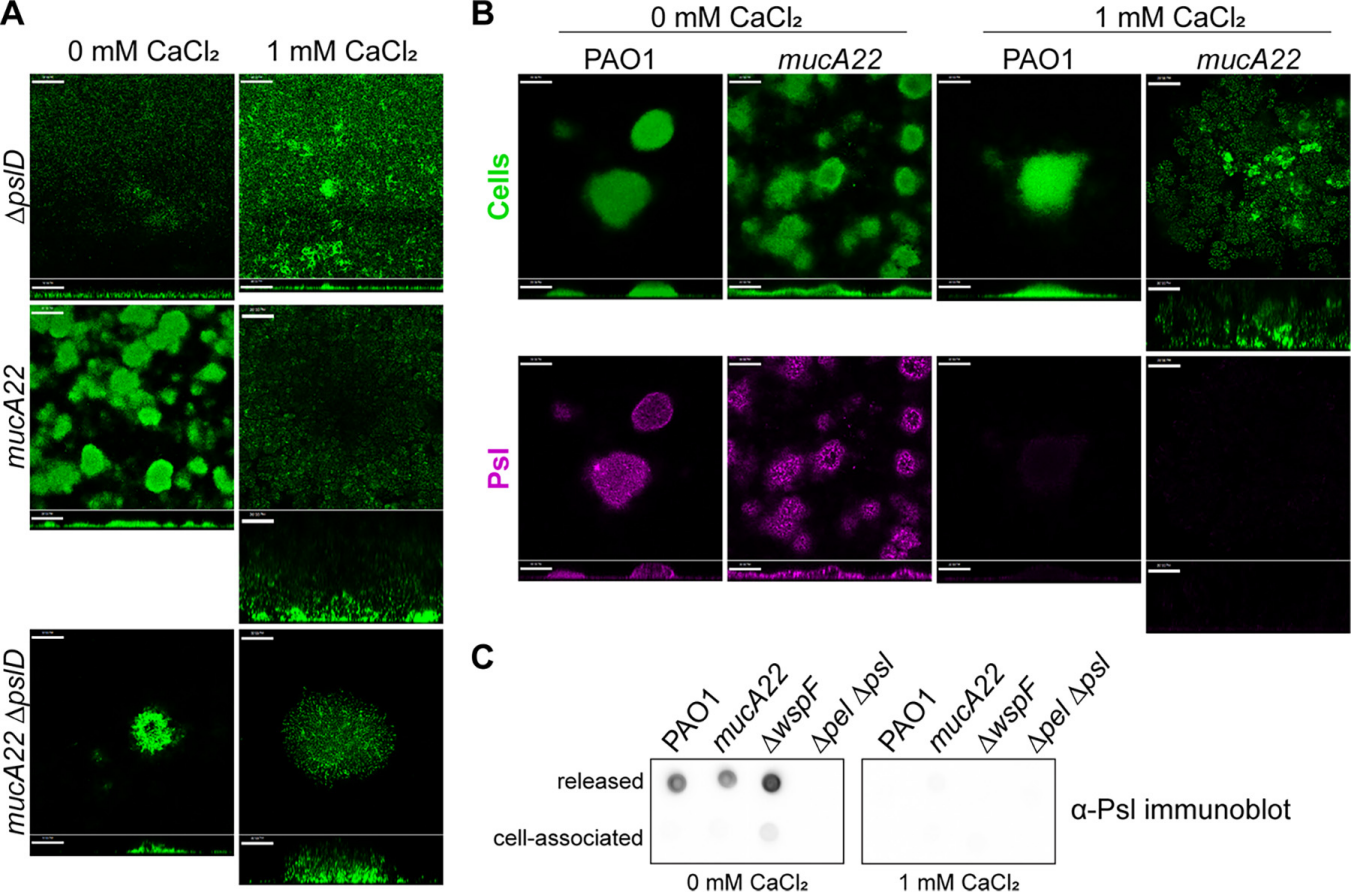
Psl is not required for the formation of calcium-cross-linked mucoid biofilms. (A) Biofilm growth of PAO1 strains deficient in Psl production (Δ*pslD*). Representative confocal images are from biofilms cultivated for 72 h under continuous-flow conditions. Cells are constitutively expressing GFP (pseudocolored green). Magnification, ×200. Bars, 50 μm. (B) Psl localization in nonmucoid and mucoid 72-h-old flow cell biofilms grown with and without calcium. Cells are constitutively expressing GFP (pseudocolored green). Psl was visualized using a fluorescent Psl-specific lectin (HHL-TRITC) (magenta). Horizontal cross sections (squares) from the middle of the biomass and sagittal views (rectangles) are shown. Magnification, ×200. Bars, 50 μm. (C) Anti-Psl immunoblotting performed on released and cell-associated fractions of statically grown biofilms. Cultures were grown for 24 h at 37°C in 5 mL of EPRI medium in wells of a 6-well tissue culture plate.

EPSs are another critical structural component of the P. aeruginosa biofilm matrix. Previous work suggested that Psl is crucial for the structure of mucoid biofilms, although the impact of calcium-cross-linked alginate was not considered (25, 35). Therefore, we examined the importance of Psl to mucoid biofilms grown with elevated calcium. We found that in the absence of elevated calcium, both PAO1 Δ*pslD* and PAO1 *mucA22* Δ*pslD* did not progress past a thin layer of attached cells (Fig. 4A). This is consistent with previous studies investigating the role of Psl in nonmucoid biofilms (36). However, PAO1 *mucA22* Δ*pslD* cells grown with 1 mM CaCl_2_ were able to form gelled biofilm structures after 72 h of growth (Fig. 4A). There was a defect in initial attachment for the Psl-deficient strains, which appears to have translated to the slower establishment of calcium-cross-linked biofilms. These results are consistent with a previous study demonstrating that Psl is crucial for the attachment and formation of aggregate structures in nonmucoid and mucoid P. aeruginosa (25). However, Psl does not appear to play an important role in later stages for mucoid strains grown with elevated calcium.

Psl production in mucoid biofilms was further investigated by using Psl-specific lectin microscopy staining. PAO1 and PAO1 *mucA22* grown without additional calcium contained abundant Psl, largely localized at the periphery of aggregates, as has been previously demonstrated (Fig. 4B) (37). In contrast, in medium containing 1 mM CaCl_2_, biofilms exhibited decreased Psl staining, particularly in the mucoid strain (Fig. 4B). Anti-Psl immunoblotting demonstrated that when biofilms were grown statically with excess calcium, Psl production was greatly reduced in both PAO1 and PAO1 *mucA22* (Fig. 4C; Fig. S4A). Interestingly, static growth in EPRI medium lacking calcium resulted in the production of primarily the released form of Psl, while the addition of 1 mM CaCl_2_ seemingly eliminated all Psl production irrespective of the strain. This was especially surprising in PAO1 Δ*wspF*, which is known to produce abundant Psl (38). We confirmed that calcium does not interfere with the recognition of Psl by the anti-Psl antibody (Fig. S4B). Investigation into the mechanism underlying how calcium impacts Psl levels is ongoing.

The EPS Pel is also produced in PAO1 biofilms, although it is less important structurally than Psl (13, 14). We found that Pel-deficient mucoid strains grown with calcium still form thick, calcium-gelled biofilms identical to those of Pel-sufficient mucoid strains (Fig. S5A). Consistent with these observations, no Pel was detected by immunoblotting in statically grown mucoid biofilm cultures, regardless of the presence of calcium (Fig. S5B). This indicates that Pel is not present in calcium-cross-linked alginate biofilms produced by the mucoid strain. These data demonstrate that Psl and Pel are not required for, and are largely absent in, calcium-gelled mucoid biofilms.

### Mucoid biofilm cells lack the high global c-di-GMP levels characteristic of nonmucoid biofilms.

**FIG 5:**
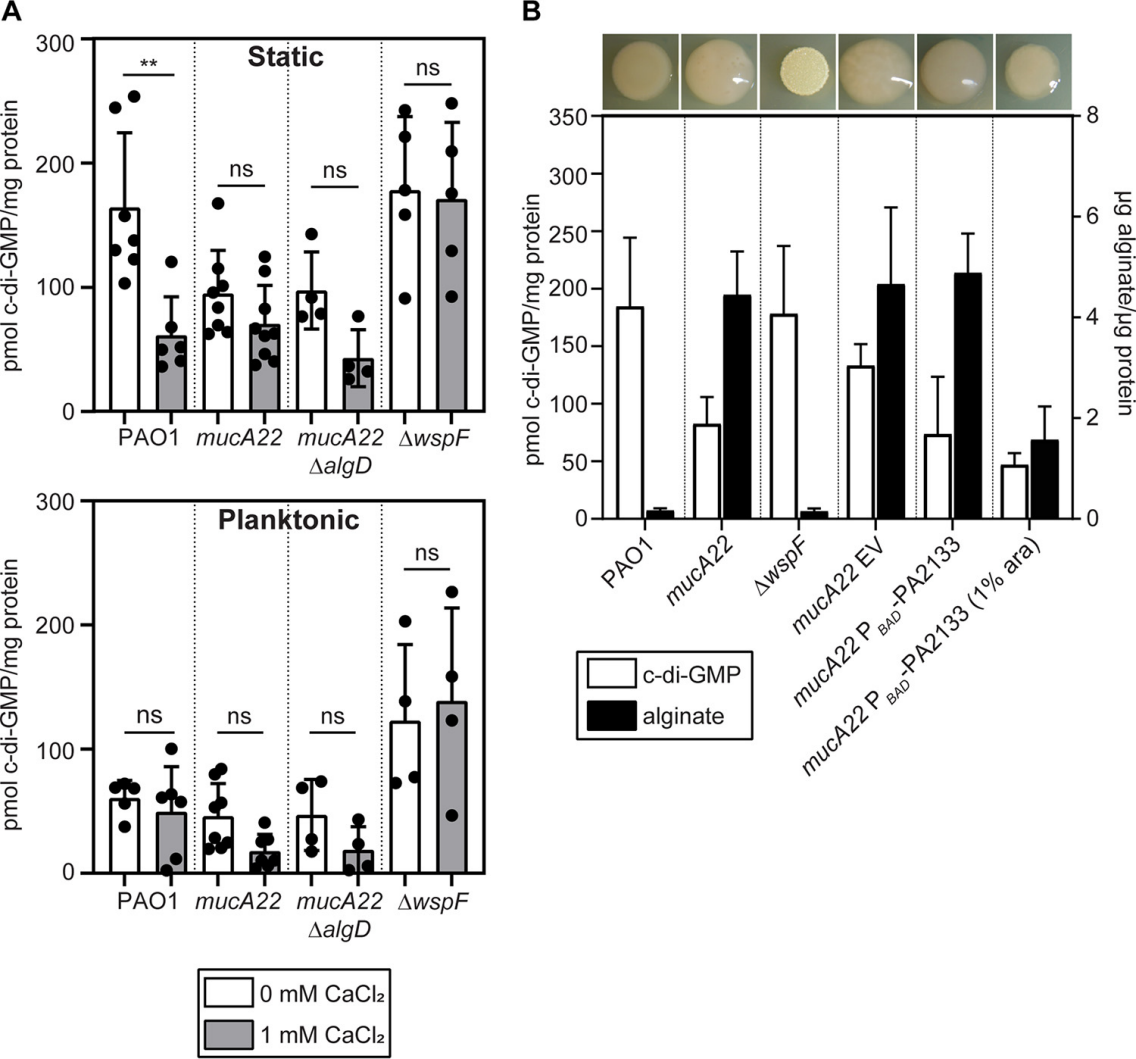
Elevated c-di-GMP is not required for alginate production in mucoid P. aeruginosa. (A) c-di-GMP quantified from cultures statically grown in EPRI medium for 24 h at 37°C with or without added calcium (top) and planktonic cultures grown to early stationary phase (OD_600_ = 0.8) in EPRI medium with or without added calcium at 37°C with shaking (bottom). **, *P* < 0.01; ns, not significant (*P* > 0.05) (by an unpaired, two-tailed *t* test). (B) c-di-GMP quantified from cultures statically grown in EPRI medium for 24 h at 37°C plotted on the left *y* axis (white bars) (PAO1, *mucA22*, and Δ*wspF* data from panel A are recapitulated here) and alginate from colonies grown on EPRI agar for 36 h at 37°C plotted on the right *y* axis (black bars). Alginate was quantified using the uronic sugar carbazole assay. EV indicates the empty vector (pJN105). P*_BAD_*-PA2133 indicates the presence of the phosphodiesterase PA2133 under arabinose-inducible control on the pJN105 backbone. Representative colonies at the time of collection for alginate measurement are shown above the corresponding bars.

The secondary messenger c-di-GMP promotes the production of biofilm matrix components such as Psl, Pel, and CdrA (10, 39). In nonmucoid strains, high global c-di-GMP levels are a recognized feature of biofilm growth (38). However, c-di-GMP has not been directly quantified in mucoid strains, and the limited expression of Psl, Pel, and CdrA in calcium-cross-linked mucoid biofilms led us to question its importance. To start, we quantified c-di-GMP in both biofilm- and planktonically grown mucoid and nonmucoid strains (Fig. 5A). As expected, independent of calcium, planktonic c-di-GMP levels were relatively low in PAO1, PAO1 *mucA22*, and PAO1 *mucA22* Δ*algD* and high in the PAO1 Δ*wspF* strain (in which the diguanylate cyclase WspR is constitutively active) (Fig. 5A). The most surprising result was that biofilms of the mucoid strain produced lower levels of c-di-GMP regardless of the presence of calcium (Fig. 5A). PAO1 *mucA22* Δ*algD* had similarly low levels of c-di-GMP regardless of calcium, indicating that low c-di-GMP is not due to the presence or absence of alginate but is presumably being mediated by AlgU regulation. Growth in calcium-containing medium decreased c-di-GMP levels in PAO1 biofilms, which may be the effect of decreased Psl observed under this condition since released Psl can serve as a signal to stimulate higher c-di-GMP levels (39).

The production of alginate is also linked to c-di-GMP-mediated control through positive allosteric interactions with the alginate biosynthetic protein Alg44 (40). However, the low c-di-GMP levels observed in mucoid biofilms suggested that elevated c-di-GMP is not required for alginate production as it is for Psl and Pel. We then quantified alginate produced by these strains and found that even artificially lowering c-di-GMP through the induction of the P. aeruginosa phosphodiesterase PA2133 was unable to fully eliminate alginate production (Fig. 5B, black bars). The induction of PA2133 modestly reduced c-di-GMP levels, but colonies still maintained a mucoid appearance (Fig. 5B). Although a 2-fold decrease in alginate levels was observed in mucoid strains, over 10 times more alginate was still produced than in the nonmucoid strains. The quantification of c-di-GMP further indicates that the overexpression of PA2133, while decreasing global c-di-GMP, has a limited impact on alginate production in PAO1 *mucA22* (Fig. 5B, black bars). This is in contrast to the PAO1 Δ*wspF* strain, where the induction of PA2133 significantly decreased c-di-GMP and EPS production, resulting in a smooth colony morphotype as opposed to the rugose morphology associated with the Δ*wspF* strain (Fig. S6) (38). These data suggest that globally elevated c-di-GMP is not required for alginate production.

### Calcium-gelled mucoid biofilms can impede penetration by antibiotics.

**FIG 6:**
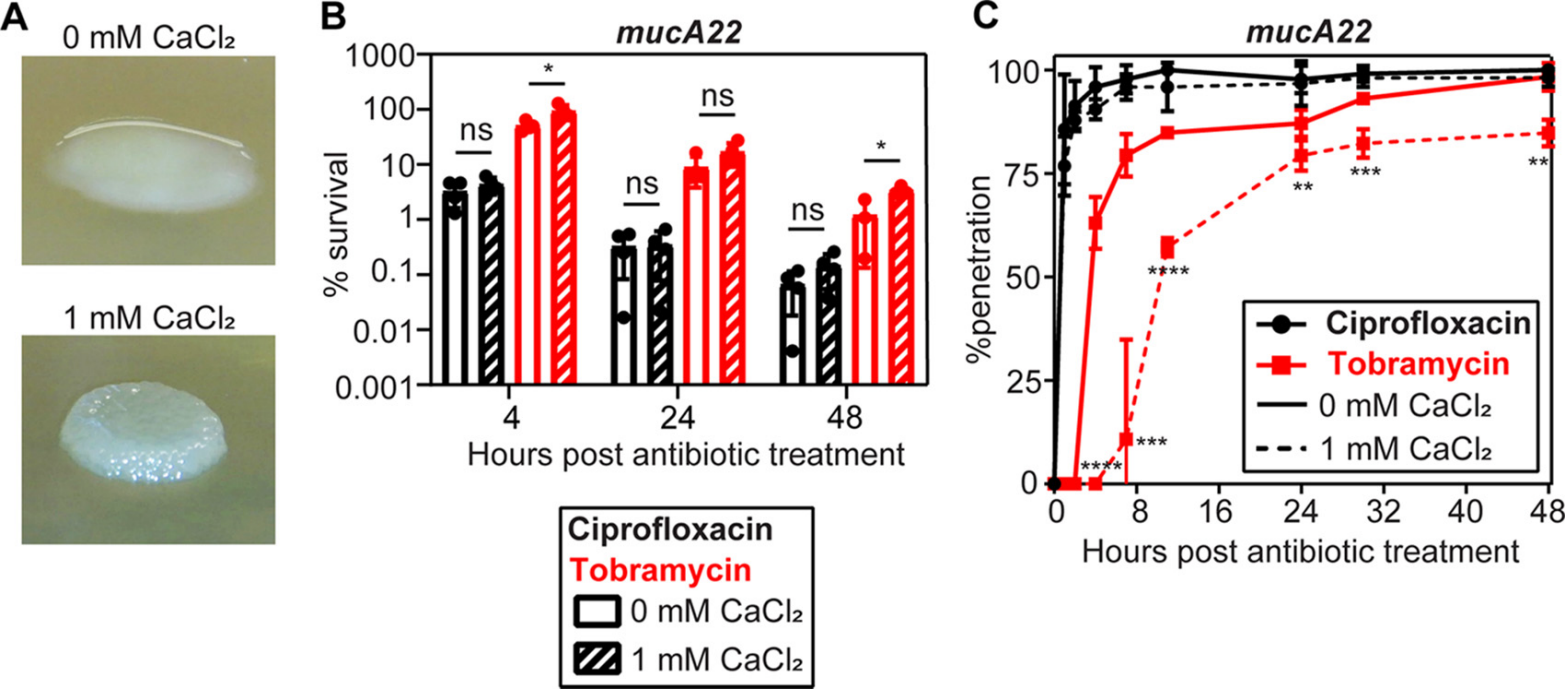
Penetration of tobramycin through mucoid biofilms. (A) Morphology of representative colony biofilms of PAO1 *mucA22* used in antibiotic penetration experiments. Planktonic cultures grown overnight in NSLB were diluted (OD_600_ = 0.1) and spotted (5 μL) onto UV-sterilized membranes placed on the agar surface. Liquid was allowed to absorb into the agar before growing cultures inverted for 24 h at 37°C. (B) Tobramycin (red) and ciprofloxacin (black) killing of *mucA22* colony biofilms. Colony biofilms were resuspended at the indicated time points after placement onto antibiotic-containing agar, serially diluted, and plated onto NSLB agar. CFU were counted after 24 h of growth at 37°C, and percent survival was calculated using CFU counts at 0 h (before antibiotic exposure) (*n* = 4). *, *P* < 0.05; ns, not significant (*P* > 0.05) (by an unpaired, two-tailed *t* test). (C) Percent penetration of antibiotic through colony biofilms grown on agar supplemented with calcium and antibiotic. Colony biofilms grown on a membrane (0.22 μm) for 24 h were transferred to plates containing antibiotics. Another membrane and a paper disk were placed on top of the colony, which was then transferred to antibiotic-containing agar for up to 48 h. Antibiotic penetration was measured by placing paper disks onto a lawn of antibiotic-sensitive E. coli and measuring the zones of inhibition. Percent penetration was calculated by dividing the diameter of the zone produced by a disk from the top of a colony biofilm by the diameter of the zone produced by a disk placed on top of two membranes only from the same time point, multiplied by 100%. Ciprofloxacin is shown in black, and tobramycin is in red. Dashed lines indicate the presence of 1 mM CaCl_2_ in the agar throughout the experiment (*n* = 4). **, *P* < 0.01; ***, *P* < 0.001; ****, *P* < 0.0001; ns, not significant (*P* > 0.05) (by an unpaired, two-tailed *t* test between tobramycin treatment of cultures grown with and without calcium). Unpaired, two-tailed *t* tests comparing 0 to 1 mM CaCl_2_ with ciprofloxacin treatment at each time point were not significant (*P* > 0.05).

Given the distinct structure and cellular arrangement in calcium-gelled alginate biofilms, we hypothesized that this would impact the ability of antibiotics to penetrate the biofilm. To test this, we grew mucoid (PAO1 *mucA22*) and nonmucoid (PAO1 *mucA22* Δ*algD*) colony biofilms on agar lacking or supplemented with 1 mM CaCl_2_. PAO1 *mucA22* colony biofilms grown without extra calcium had the expected slimy, mucoid appearance, while calcium-gelled mucoid colonies formed more prominent, rigid structures (Fig. 6A). PAO1 *mucA22* Δ*algD* colony biofilms had a smooth, nonmucoid morphology, unaffected by the addition of calcium (Fig. S7A). Ciprofloxacin rapidly killed cells in both nonmucoid and mucoid colony biofilms, regardless of calcium (Fig. 6B; Fig. S7B). Calcium also did not influence tobramycin killing of nonmucoid colony biofilm cells (Fig. S7B). However, mucoid cells in a calcium-gelled colony biofilm were significantly protected throughout the course of tobramycin challenge compared to mucoid colony biofilms grown without extra calcium (Fig. 6B). Calcium-gelled alginate colony biofilms also greatly slowed the penetration of tobramycin through the colony in comparison to mucoid biofilms grown without calcium (Fig. 6C). As previously reported, ciprofloxacin rapidly penetrated all colony biofilms (Fig. 6C; Fig. S7C) (18). Calcium did not affect tobramycin penetration in nonmucoid biofilms (Fig. S7C).

It has been speculated that the negative charge of alginate adsorbs and slows the transit of positively charged antibiotics such as tobramycin (18). To test this, we assessed the ability of two similarly sized charged nonantibiotic molecules to penetrate colony biofilms (fluorescein [negatively charged] and rhodamine B [positively charged]). However, there were limited differences in the rates of fluorescein and rhodamine B penetration through ungelled and gelled mucoid biofilms (Fig. S8A). Penetration by these molecules was no different between mucoid and nonmucoid strains and appeared unaffected by the presence of calcium (Fig. S8A and B). These data suggest that factors beyond simple charge interactions are responsible for the slower penetration of tobramycin through calcium-gelled alginate biofilms.

### Evidence that calcium-cross-linked alginate biofilms are present in CF airways.

**FIG 7:**
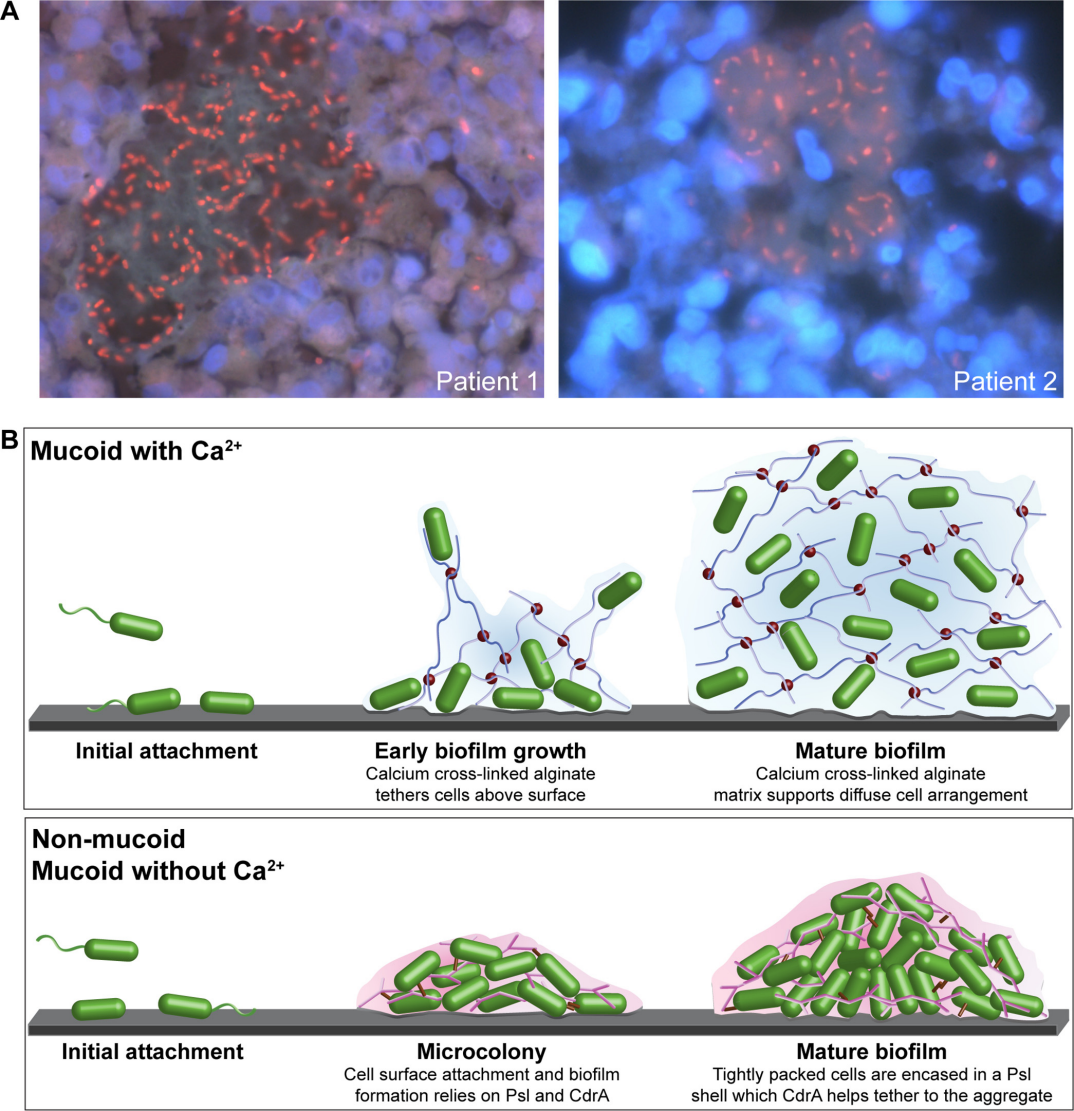
A model for P. aeruginosa biofilm formation. (A) *In vivo* mucoid biofilms. Shown are representative mucoid P. aeruginosa biofilms within mucus plugs of explanted CF lungs. P. aeruginosa (red) was visualized by a Texas Red-labeled, P. aeruginosa-specific peptide nucleic acid (PNA) probe and counterstained with 4′,6-diamidino-2-phenylindole (DAPI) to visualize surrounding polymorphonuclear leukocytes (PMNs) (blue). Patient 1 (CF 204) and patient 2 (CF 159) details are in Table S2 in the supplemental material. Magnification, ×1,000. (B) Models of PAO1 P. aeruginosa biofilm development. (Top) When grown under high-calcium conditions, calcium-cross-linked alginate tethers mucoid cells together and to surfaces. A mature mucoid biofilm is comprised of a calcium-cross-linked alginate matrix in which cells are diffusely suspended (Fig. 1C). In contrast to nonmucoid PAO1 strains, CdrA, Psl, and Pel do not play a major role in the formation or maintenance of these calcium-cross-linked alginate biofilm structures. (Bottom) Nonmucoid and mucoid strains grown without high calcium rely on CdrA and Psl for attachment to surfaces and the structural integrity of mature biofilm aggregates. These models are not drawn to scale. Cells are green capsules. Alginate polymers are indicated by the blue lines, and calcium ions are small red spheres. Psl polymers are branched pink lines, and CdrA is indicated by small brown cylinders. The cartoon model was constructed using Adobe Illustrator 25.4.1.

Calcium concentrations within CF airways have been estimated to be 1 to 5 mM (33, 34, 41). This suggests that mucoid P. aeruginosa strains have the potential to produce calcium-cross-linked alginate biofilms during chronic CF airway infections. Previous work has demonstrated that P. aeruginosa biofilm aggregates producing a Psl- and Pel-rich biofilm matrix are present in CF sputum and lung samples and are characterized by tightly packed bacterial aggregates (42, 43). We therefore examined explanted CF lung samples for the presence of individual cell suspensions characteristic of *in vitro* calcium-cross-linked alginate gels. These CF patients had previously tested culture positive for both mucoid and nonmucoid P. aeruginosa (Table S2). We observed that the bacterial cells, while clearly within a cohesive aggregate of matrix material, also appeared to be discretely spaced from one another (Fig. 7A). While the concentration of calcium was not measured in these samples, nor were these exact aggregates determined to exclusively contain mucoid cells, the organization of diffusely spaced cells is strikingly similar to that of *in vitro*-cultivated calcium-gelled alginate biofilms (Fig. 1A and C). These data suggest that our description of mucoid biofilm formation in a high-calcium environment may be relevant to CF lung infections.

## DISCUSSION

Collectively, our data suggest that mucoid P. aeruginosa employs a calcium-dependent program of biofilm formation that is distinct from the c-di-GMP central paradigm (Fig. 7B). Under these circumstances, mucoid P. aeruginosa uses alginate as the primary biofilm matrix component. Previous reports suggested that, like nonmucoid strains, mucoid strains rely upon CdrA and Psl for structural robustness (10, 25, 35). However, these studies were conducted in the absence of elevated calcium. Our study demonstrates that Psl, Pel, and CdrA are not produced in or required for a calcium-cross-linked mucoid biofilm. Instead, the calcium-gelled alginate matrix appears to rely solely on calcium-alginate cross-linking for support, although other matrix components may contribute to the maintenance of these biofilm structures. Indeed, due to the disperse arrangement of cells within the gelled alginate matrix, we hypothesize that yet-to-be-identified biofilm matrix components are interacting with the alginate polymers.

Our findings that high global c-di-GMP levels are not required for alginate overproduction were surprising as increased levels of Psl and Pel production are both dependent on elevated c-di-GMP (9). Alginate biosynthesis is reliant on a c-di-GMP binding protein, Alg44, the activity of which is likely regulated by the diguanylate cyclase MucR (40, 44). The expression of the phosphodiesterase PA2133 drastically reduces global c-di-GMP pools, resulting in pronounced effects on Psl and Pel production (38). Our data suggest that c-di-GMP controls alginate biosynthesis through spatially localized events near Alg44, which may be insensitive to the general phosphodiesterase activity of PA2133 (44). However, quantifying global c-di-GMP levels as we have done in this study cannot provide the necessary information to directly test this hypothesis.

Calcium is central to the phenotypes described in this study. Calcium-alginate interactions appear to be solely responsible for the unique biofilm structure formed by mucoid strains, as the chelation of calcium entirely disrupts these biofilms (Fig. 2). Furthermore, additional calcium has no visible effect on the development and structure of the aggregates formed by nonmucoid strains despite the decreased Psl produced under these conditions (Fig. 4B and C; see also Fig. S2 in the supplemental material). While the importance of calcium as a signal for prokaryotes is poorly characterized relative to eukaryotes, calcium may influence biofilm formation and induce virulence factor production in P. aeruginosa (28–30, 45). These effects of supplemental calcium were not specifically investigated in our study, and the intracellular effects mediated by calcium in our biofilm system are still being actively studied. For example, we show that growth in EPRI medium with 1 mM CaCl_2_ correlated with decreased CdrA and Psl production across both mucoid and nonmucoid P. aeruginosa strains. Exactly how calcium causes these changes and how the medium used contributes to these results are currently unknown.

We acknowledge that this work studied primarily pure-culture, *in vitro*-grown biofilm communities. Mucoid and nonmucoid P. aeruginosa strains are often isolated from the same chronically infected CF patient, along with a host of other microbial species (46). Work has demonstrated that *in vitro* mixed mucoid and nonmucoid P. aeruginosa communities can fulfill different but complementary functions, with each being able to protect the other from an antimicrobial to which it is otherwise susceptible (47). If and how nonmucoid P. aeruginosa strains integrate with calcium-gelled alginate biofilms in the niche of CF airways remains an actively studied area. Additionally, alginate inhibits macrophage opsonization and can prevent the interferon (IFN)-mediated clearance of biofilm-embedded P. aeruginosa (48, 49). The question of whether calcium-gelled alginate biofilms impact clearance by the immune system also remains to be addressed.

Data indicating that P. aeruginosa can form a biofilm matrix comprised solely of calcium-cross-linked alginate have serious health implications. Strains of mucoid P. aeruginosa arise over time in chronically infected individuals (15, 50). Our staining of bacterial aggregates in CF lungs illustrates that alginate-containing aggregates contain diffusely organized cells, extremely similar to the cellular arrangement observed in the *in vitro*-grown mucoid calcium-gelled biofilm structures. The penetration of tobramycin through alginate biofilms is somewhat impeded, presumably on the basis of the charge interaction between positively charged tobramycin and negatively charged alginate (18). We report here that tobramycin penetrates alginate-deficient biofilms slightly slower than it penetrates alginate biofilms grown without calcium, while its penetration of calcium-cross-linked alginate biofilms is significantly impeded (Fig. 6C; Fig. S7C). Furthermore, potential oxygen limitation within colony biofilms, although not measured in this study, may also contribute to the decreased tobramycin-mediated cell death observed in calcium-gelled biofilms (Fig. 6B) (18). This suggests that the interplay between alginate in the biofilm matrix and tobramycin is more complex than initially appreciated. Our data involving penetration by rhodamine B, a similarly sized positively charged compound, suggest that positive charge may contribute somewhat to the reduced penetration (Fig. S8A). It remains to be determined what additional factors, such as changes in mechanical stiffness or cellular spacing, could be impacting the observed slower penetration of tobramycin.

We have defined here a unique pattern of mucoid P. aeruginosa biofilm matrix development that is reliant upon calcium. This developmental program results in a biofilm with individual cells suspended in a calcium-cross-linked exopolysaccharide matrix. Traditional biofilm matrix components that are critical in nonmucoid biofilms appear to be dispensable. Our data also suggest that c-di-GMP plays a less prominent role in controlling the formation of these mucoid biofilms. We also provide evidence that this unique program of biofilm development may occur in the CF airways, which are frequently populated by mucoid P. aeruginosa. These findings highlight the versatility of P. aeruginosa in using different biofilm matrix components to produce communities with distinct structures and properties.

## MATERIALS AND METHODS

### Bacterial strains, growth conditions, and antibiotics.

Plasmids and bacterial strains used in this study are listed in Table S1 in the supplemental material. All P. aeruginosa strains were propagated at 37°C in no-salt lysogeny broth (NSLB) unless otherwise noted. Strains constitutively producing green fluorescent protein (GFP) were made by inserting *gfp* at the Tn*7* site as previously described (51). Deletions of *algD*, *pslD*, *pelA*, and *cdrA* and mutation of *mucA* were created as previously described (52). The PAO1 *mucA22* Δ*algD* strains could be complemented by the introduction of the plasmid pJN105::*algD* (which expresses *algD* under the control of an arabinose-inducible promoter) and growth with 1% l-arabinose (data not shown). The following concentrations of antibiotics were used for the selective growth of plasmid-containing strains: 50 μg/mL kanamycin and 10 μg/mL gentamicin for Escherichia coli and 30 to 100 μg/mL gentamicin and 300 μg/mL carbenicillin for P. aeruginosa. Growth curves were constructed for all P. aeruginosa strains, and no growth defects were observed. Flow cell biofilms were grown in modified EPRI medium containing 0.01% sodium lactate, 0.005% sodium succinate, 0.005% NH_4_NO_3_, 0.00019% KH_2_PO_4_, 0.00063% K_2_HPO_4_, 0.001% (vol/vol) Hunter salts, 0.1% glucose, and 0.001% l-histidine. CaCl_2_ was added to a final concentration of 1 mM (53).

### Antibodies, immunoblotting, and Western blotting.

Primary antibodies were diluted in 1% milk in Tris-buffered saline with 0.05% Tween 20 (TBST). Antibodies used were anti-Psl (1:3,000) (MedImmune) (54), anti-Pel (1:1,000) (13), and anti-CdrA (1:10,000) (11). CdrA, Pel, and Psl were blotted as previously described (11, 13, 55).

### Static biofilm growth.

Six-well plates were inoculated with 5 mL/well of a 1:20-diluted mid-log-phase culture (optical density at 600 nm [OD_600_] = 0.4 to 0.8). The plates were incubated for 24 h at 37°C. The entire culture was passed through an 18-gauge needle five times and vortexed well prior to normalization to an OD_600_ of ∼0.310. One milliliter of the normalized culture was centrifuged, and the supernatant was retained (“released” EPS or protein). The cell pellet was boiled in 0.5 M EDTA and centrifuged, and the supernatant was retained (“cell-associated” EPS). EPS samples were proteinase K treated prior to immunoblotting. The protein content was measured (Qubit protein assay kit; Thermo Fisher), and samples were normalized to total protein. A total of 0.6 μg of total protein was loaded for each sample. Purified EPS was prepared as previously described (8).

### Flow cell biofilms and confocal microscopy.

P. aeruginosa strains were grown in NSLB until mid-log phase (OD_600_ = 0.4 to 0.8) and then diluted to an OD_600_ of 0.05 in EPRI medium. Flow cell chambers were inoculated with the diluted cultures and incubated inverted for 1 h before initiating flow. Biofilms were continuously supplied with fresh medium at a rate of 10 mL/h. Flow cells were grown at room temperature and imaged at 24, 48, and 72 h. Biofilms were imaged using a Zeiss LSM 800 confocal laser scanning microscope (Carl Zeiss, Jena, Germany), and image processing was done using Volocity software (Improvision). Hippeastrum hybrid lectin conjugated with tetramethylrhodamine isothiocyanate (HHL-TRITC) was used to visualize Psl (100 μg/mL; GlycoMatrix) as previously described (8). Microscopic observation indicated that this staining procedure did not change the overall biofilm morphology.

### EGTA treatment of biofilms.

EGTA (Sigma) was added to 0 mM CaCl_2_ EPRI growth medium (final concentration, 5 mM [pH 7.0]). EGTA-containing medium was connected to a channel containing the 48-h biofilm to be imaged, and flow was resumed. Images of the same field of view were acquired while flowing with EGTA medium to keep the concentration constant.

### Aggregation assay.

The CdrA-mediated aggregation assay was performed as previously described (12). d-Mannose, l-rhamnose, alginic acid, and sodium alginate from Sigma-Aldrich and uronic acids from Carbosynth LLC were used at 5 mg/mL. Bacterial alginate (Dextra Laboratories Ltd.) was used at 0.5 mg/mL due to poor solubility. Aggregation was visually evaluated and then measured by optical density readings taken at 600 nm. Percent relative aggregation was calculated by taking the difference between the OD_600_ of the P_*BAD*_-*cdrAB* strain and that of its corresponding vector control strain, dividing by the OD_600_ of the vector control strain, and multiplying by 100%.

### c-di-GMP quantification.

c-di-GMP was quantified from cultures grown in EPRI medium at 37°C (static growth for 24 h and planktonic growth with shaking until the OD_600_ reached 0.8). c-di-GMP was extracted as previously described (39, 56), using 2-chloro-AMP as an internal standard. All experiments were performed on ≥3 independent replicates. Briefly, c-di-GMP was extracted from pelleted cells by incubation with 70% perchloric acid on ice for 1 h. The supernatant was retained and neutralized using potassium bicarbonate. Liquid chromatography-tandem mass spectrometry (MS/MS) measurements were performed using an Acuity ultraperformance liquid chromatography (UPLC) system with a Synergi 4μ Hydro-RP 80A column and a C_18_ guard cartridge (Phenomenex) on a Premier XL triple-quadrupole electrospray mass spectrometer (Waters). The *m/z* 691 > 152 transition was used for c-di-GMP, and the *m/z* 382 > 170 transition was used for 2-chloro-AMP. The cone voltages and collision energies were 40 V/30 eV and 35 V/20 eV, respectively. Fifteen microliters of each sample was injected, and the area under the curve of the c-di-GMP channel signal (retention time of 1.6 min) was divided by the area under the curve of the 2-chloro-AMP signal (retention time of 2.1 min). A standard curve of 0 nM to 100 nM c-di-GMP containing 2-chloro-AMP was used to quantify c-di-GMP for all samples. The c-di-GMP concentration was normalized to the total protein concentration.

### Alginate quantification.

Alginate was quantified using the uronic acid carbazole assay with several modifications (57). Thirty microliters of a mid-log-phase culture was spotted onto EPRI medium and EPRI medium with 1% arabinose agar and grown at 37°C for 24 h. The entire colony was collected into 500 μL of 1 M NaCl, vortexed extensively, and then centrifuged. The supernatant was used for alginate quantification, and the cell pellet was used for protein quantification. Samples were added in triplicate to a 96-well polypropylene plate (50 μL). A total of 25 mM sodium tetraborate in concentrated sulfuric acid (200 μL) was added. The plate was covered and gently shaken for 3 min before incubation uncovered at 60°C for 20 min. The plate was cooled to room temperature for 15 min before adding 0.125% carbazole in absolute ethanol (50 μL). The plate was covered and gently shaken for 3 min before incubation uncovered at 60°C for 20 min. The plate was cooled to room temperature for 15 min before transferring 250 μL of each sample into a transparent flat-bottomed 96-well plate and reading at 550 nm on a microplate reader (CLARIOstar Plus microplate reader; BMG Labtech). The concentration of alginate was determined using known dilutions of sodium alginate to generate a standard curve fit with a linear equation.

### Colony biofilms and antibiotic penetration.

Colony biofilms were grown as previously described, with slight modifications (18). Five microliters of each culture (OD_600_ = 0.1) was spotted onto a UV-sterilized polycarbonate membrane (25 mm; GVS Filter Technology) on NSLB or NSLB with 1 mM CaCl_2_ (NSLBC) agar. Plates were grown at 37°C for 24 h. To assess antibiotic penetration, another polycarbonate membrane (13 mm; GVS Filter Technology) was added, and a wetted paper disk was then placed on top of the 24-h colony biofilms. This assembly was then moved to NSLB or NSLBC containing tobramycin (50 μg/mL), ciprofloxacin (10 μg/mL), fluorescein (50 μg/mL), or rhodamine B (50 μg/mL). The paper disks were removed at each time point and stored at 4°C. The antibiotic disks were placed onto Mueller-Hinton agar spread with a dilute culture of E. coli (ATCC 25922). The diameters of the zones of inhibition were measured after 8 h of growth at 37°C. Percent penetration was calculated by dividing the diameter of the zone produced by a disk from the top of a colony biofilm by the diameter of the zone produced by a disk placed on top of two membranes only from the same time point, multiplied by 100%. The fluorescent disks were placed into 225 μL of phosphate-buffered saline (PBS), vortexed for 1 min, and then allowed to equilibrate for 30 min prior to measuring fluorescence (fluorescein ex/em: 483/530 and rhodamine B ex/em: 550/605) (CLARIOstar Plus microplate reader; BMG Labtech). The concentration of the penetrating fluorophore was determined using dilutions of fluorescein and rhodamine B to generate a standard curve fit with a linear equation. Sterile apparatuses (two membranes and a paper disk without a colony biofilm) were assembled and assayed concurrently.

### CF patient samples and visualization by fluorescence *in situ* hybridization using peptide nucleic acid probes (PNA FISH).

Tissue samples from explanted lungs from a CF patient chronically infected with P. aeruginosa undergoing double-sided lung transplantation were obtained immediately upon the removal of each lung. Patient details are provided in Table S2. The lungs were collected with the acceptance of the patients and in accordance with the biomedical project protocol (KF-01278432) approved by the Danish scientific ethical board. The patients had chronic P. aeruginosa infection harboring both mucoid and nonmucoid bacteria (as identified in sputum prior to transplantation). Samples were immediately fixed in PBS with 4% paraformaldehyde. The tissue was embedded in paraffin, sectioned (4 mm), and mounted onto glass slides. Samples were deparaffinized, hybridized, and visualized as described previously (43).

### Statistical analysis.

All data were analyzed and plotted using GraphPad Prism version 9.2.0 for Windows (GraphPad Software, San Diego, CA, USA [www.graphpad.com]). Data are plotted as the means ± standard deviations (SD) unless otherwise indicated in the associated figure legends. Unpaired, two-tailed *t* tests or one-way analyses of variance (ANOVAs) were performed as noted in the figure legends.
