# Supplementary material for: Mucoid Pseudomonas aeruginosa Can Produce Calcium-Gelled Biofilms Independent of the Matrix Components Psl and CdrA
Source: J Bacteriol. 2022 Apr 13;204(5):e00568-21. doi: 10.1128/jb.00568-21 (PMC9112934; doi:10.1128/jb.00568-21)
Supplement: Supplemental file 1 — Tables S1-S2 and Fig. S1-S8. Download jb.00568-21-s0001.pdf, PDF file, 4.6 MB [file jb.00568-21-s0001.pdf]

Table S1. Plasmids and strains used

**Description**	**Source**
Plasmids used	
Conjugation donor, pDONRPEX18Gm::*mucA22*	This study
Conjugation donor, pDONRPEX18Gm::Δ*algD*	This study
Conjugation donor, pDONRPEX18Gm::Δ*cdrA*	(1)
Conjugation donor, pEX18Gm::Δ*pslD*	(2)
Conjugation donor, pENTRPEX18Gm::Δ*pelA*	JJ Harrison
Mating helper strain, pRK2013	(3)
pJN105: araC-P_{BAD} cassette cloned in pBBR1MCS-5; Gm^r	(4)
pJN105::PA2133: PA2133 cloned into pJN105; Gm^r	(4)
pJN105::*algD*: *algD* cloned into pJN105; Gm^r	This study
pMJT-1	(4)
pMJT-1::*cdrAB*: *cdrAB* operon cloned under arabinose-inducible promoter	(4)

**Strains**	**Description**	**Source**
Escherichia coli		
E. coli ATCC®25922™	FDA strain Seattle 1946 [DSM 1103, NCIB 12210]	www.atcc.org
Pseudomonas aeruginosa		
PAO1	Wild-type laboratory strain	(5)
mucA22	PAO1 with a chromosomal replacement of *mucA22*, in which bp 430 of *mucA* is deleted resulting in an early stop codon (6)	(7)
Δ*algD*	PAO1 with an in-frame deletion of *algD* (Δ*algD*)	This study
mucA22 Δ*algD*	PAO1 *mucA22* Δ*algD*	This study
PAO1::*gfp*	PAO1 with tagged with promoterless gfp integrated at the att Tn7 site (Tn7::*gfp*)	This study
mucA22::*gfp*	PAO1 *mucA22* Tn7::*gfp*	This study
mucA22 Δ*algD*::*gfp*	PAO1 *mucA22* Δ*algD* Tn7::*gfp*	This study
Δ*cdrA*::*gfp*	PAO1 with an in-frame deletion of *cdrA* (Δ*cdrA*), Tn7::*gfp*	This study
mucA22 Δ*cdrA*::*gfp*	PAO1 *mucA22* Δ*cdrA*, Tn7::*gfp*	This study
mucA22 Δ*algD* Δ*cdrA*::*gfp*	PAO1 *mucA22* Δ*algD* Δ*cdrA* Tn7::*gfp*	This study
Δ*pslD*::*gfp*	PAO1 with an in-frame deletion of *pslD* (Δ*pslD*), Tn7::*gfp*	This study
mucA22 Δ*pslD*::*gfp*	PAO1 *mucA22* Δ*pslD*, Tn7::*gfp*	This study
mucA22 Δ*algD* Δ*pslD* ::*gfp*	PAO1 *mucA22* Δ*algD* Δ*pslD* Tn7::*gfp*	This study
Δ*pelA*::*gfp*	PAO1 with an in-frame deletion of *pelA* (Δ*pelA*), Tn7::*gfp*	This study
mucA22 Δ*pelA*::*gfp*	PAO1 *mucA22* Δ*pelA*, Tn7::*gfp*	This study
mucA22 Δ*algD* Δ*pelA*::*gfp*	PAO1 *mucA22* Δ*algD* Δ*pelA* Tn7::*gfp*	This study
PAO1 pMJT-1	PAO1 with plasmid pMJT-1	(4)
PAO1 pMJT-1::*cdrAB*	PAO1 with plasmid pMJT-1::*cdrAB*	(4)

PAO1 pJN105	PAO1 with plasmid pJN105	This study
PAO1 pJN105::PA2133	PAO1 with plasmid pJN105::PA2133	This study
PAO1 *mucA22* pJN105	PAO1 *mucA22* with plasmid pJN105	This study
PAO1 *mucA22* pJN105::PA2133	PAO1 *mucA22* with plasmid pJN105::PA2133	This study
PAO1 Δ*wspF* pJN105	PAO1 Δ*wspF* with plasmid pJN105	This study
PAO1 Δ*wspF* pJN105::PA2133	PAO1 Δ*wspF* with plasmid pJN105::PA2133	This study
PAO1 *mucA22* Δ*algD* pJN105::*algD*	PAO1 *mucA22* Δ*algD* with plasmid pJN105::*algD*	This study
PAO1 *mucA22* Δ*algD::gfp* pJN105::*algD*	PAO1 *mucA22* Δ*algD::gfp* with plasmid pJN105::*algD*	This study
Δ*wspF*	PAO1 Δ*wspF*	(8)
Δ*wspF* Δ*pel*	PAO1 Δ*pelA* Δ*wspF*	(9)
Δ*wspF* Δ*psl*	PAO1 Δ*pslBCD* Δ*wspF*	(9)
Δ*pel* Δ*psl*	PAO1 Δ*pelA* Δ*pslBCD*	(4)
Δ*pel* Δ*psl* Δ*cdrA*	PAO1 Δ*pelA* Δ*pslBCD* Δ*cdrA*	CR Reichhardt

Table S2. Summary of explanted lungs from cystic fibrosis patients who received new lungs due to respiratory failure caused by chronic *P. aeruginosa* lung infection

**Patient ID**	CF 204, explanted lung	CF 159, explanted lung
**Gender**	Male	Female
**Age**	41 years	30 years
**Duration of chronic *P. aeruginosa* infection**	29 years	30 years
**Bacteriological evaluation**	Mucoid *P. aeruginosa*, biofilms in respiratory (few) and conductive zones (many), non-mucoid also in conductive zone	Mucoid *P. aeruginosa*, biofilms in respiratory (few) and conductive zones (many), non-mucoid also in conductive zone
**No. of precipitating *P. aeruginosa* antibodies (normal: 0-1)**	46	46
**No. of precipitating antibodies against *S. aureus* (Sa), *H. influenzae* (Hi), (normal 0-2)**	Sa: 3, Hi: 8	Sa: 2, Hi: 8
**Lung pathology**	End stage lung parenchyma with severe bronchiectasis with acute and chronic inflammation and purulent secret stagnation. Severe peribronchial fibrosis and focal organizing pneumonia.	End stage lung parenchyma with bronchiectasis with acute and chronic inflammation and purulent secret stagnation. Focal bronchopneumonia with organization, loss of alveolated tissue and fibrous scarring.

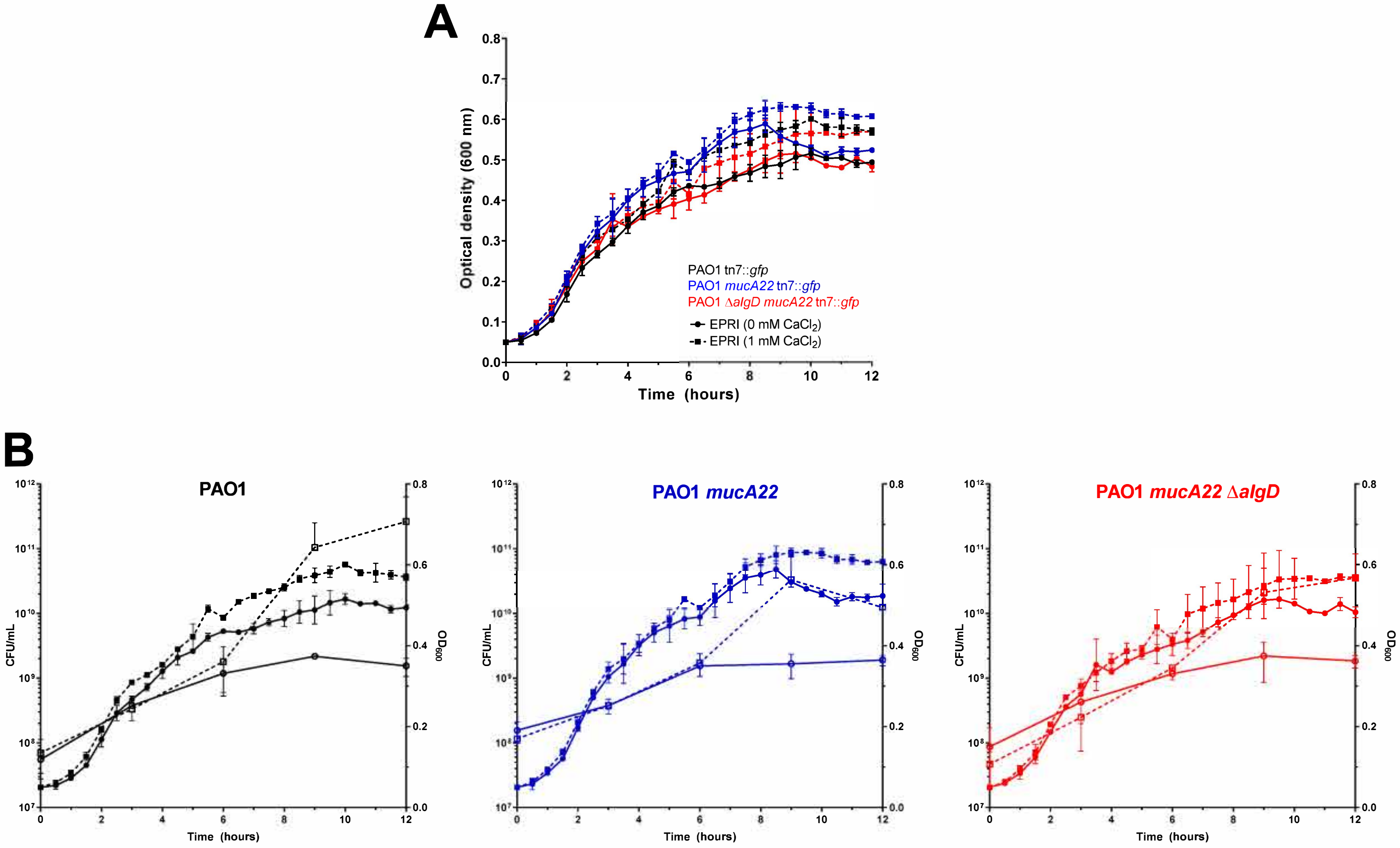

Supplemental Figure 1. Growth of frequently used *P. aeruginosa* strains. PAO1 tn7::*gfp* (black), PAO1 *mucA22* tn7::*gfp* (blue), and PAO1 *mucA22* Δ*algD* tn7::*gfp* (red). Cultures grown in 50 mL of EPRI medium (with or without added calcium) in 250 mL baffled flasks at 37°C with shaking at 225 rpm. (A) Optical density measurements (600 nm) taken every 30 min for 12 h. (B) Overlaid OD_{600} (right y-axis) and CFU/mL (left y-axis) taken at noted time points. CFU measurements were obtained by serially diluting cultures and plating onto NSLB, growing at 37°C for 18 h before counting colonies.

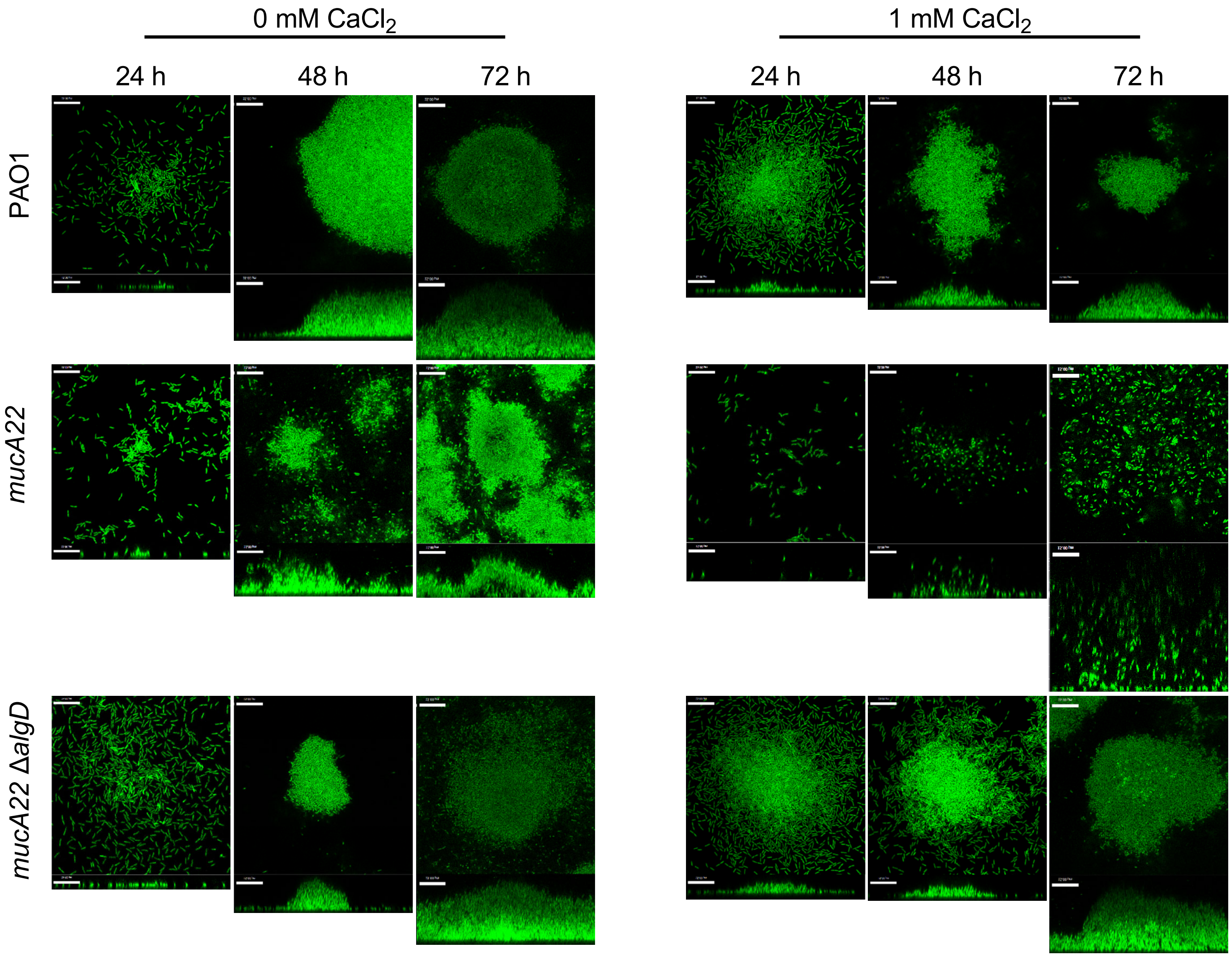

Supplemental Figure 2. Flow cell biofilm growth of mucoid and non-mucoid strains over time. Representative confocal images are from PAO1, PAO1 *mucA22*, and PAO1 *mucA22 ΔalgD* biofilms cultivated up to 72 h under continuous flow conditions with or without additional calcium added to the growth medium. Biofilms were imaged at 24, 48, and 72 h post-inoculation of the flow cell chamber. Cells are expressing GFP (pseudo-colored green) under control of a constitutively active promoter. Horizontal cross-sections (square) from the middle of the biomass and sagittal views (rectangle) are shown. Magnification used was 630X. Scale bars represent 15 µm.

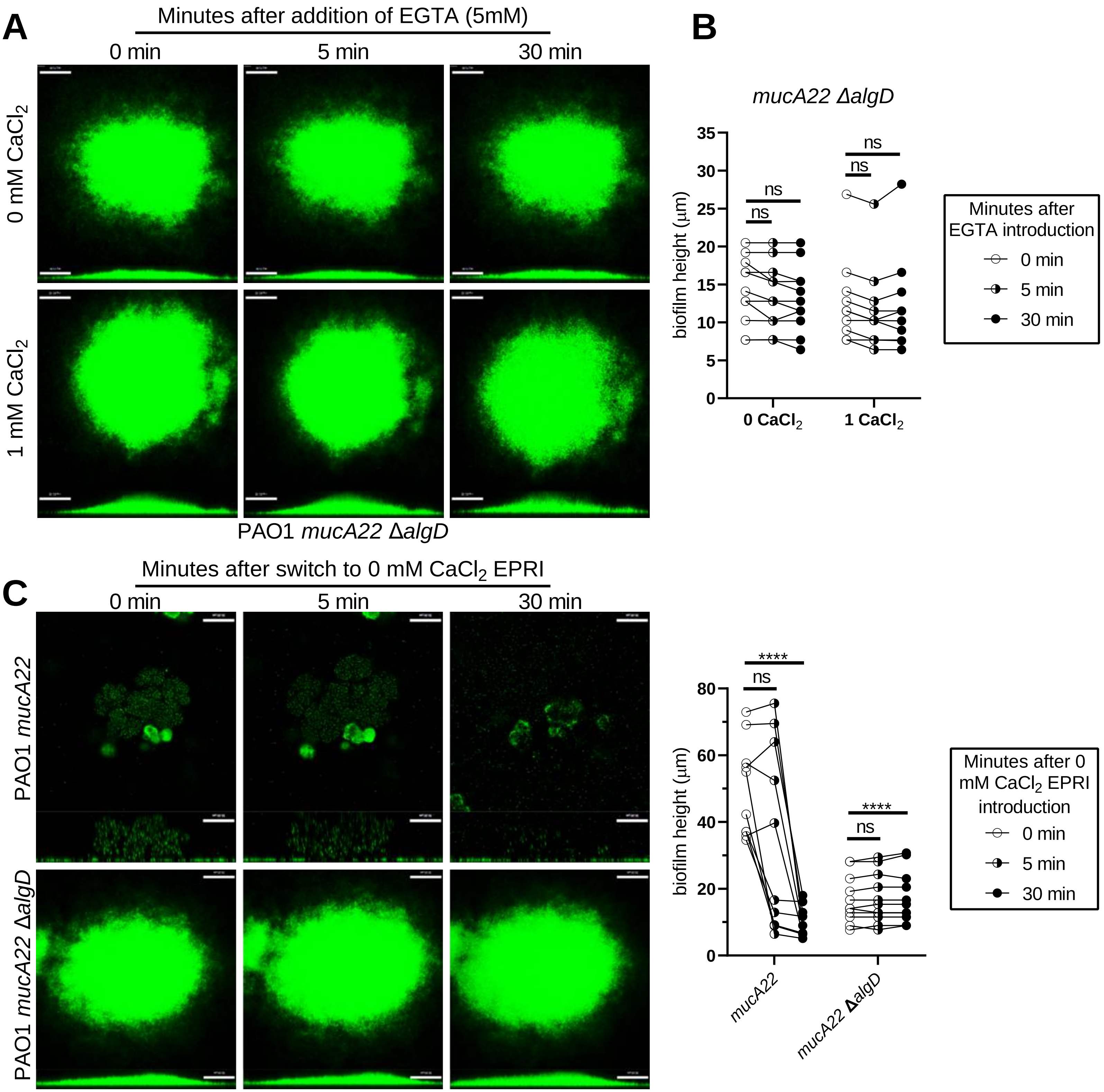

Supplemental Figure 3. Calcium chelation does not affect non-mucoid biofilm structure. (A) Representative confocal images are from PAO1 *mucA22* Δ*algD* biofilms cultivated for 48 h in flow cells prior to addition of EGTA (5 mM). Images were captured at 0, 5, and 30 min after initial introduction of EGTA (5 mM in EPRI medium containing 0 mM $CaCl_2$ supplied continuously). (B) Quantification of biofilm height (in µm) measured from base of the biofilm to its highest point at 0, 5, and 30 min of EGTA treatment demonstrates that non-mucoid biofilms do not lose height due to EGTA treatment. Quantified images are from three biological replicates with 1-2 biofilms from 3-4 images measured per condition per replicate, n=3. Each line represents a single aggregate measured at each time point. Unpaired, two-tailed t-test; ns, not significant, P>0.05. (C) PAO1 *mucA22* and *mucA22* Δ*algD* biofilms grown in 1 mM $CaCl_2$ EPRI for 48 h were switched to EPRI with 0 mM $CaCl_2$ and images were captured at 0, 5, and 30 min afterwards. Biofilm height was measured at these time points, as described in (B) ****, P<0.0001. (A,C) Cells are expressing GFP (pseudo-colored green) under control of a constitutively active promoter. Horizontal cross-sections (square) from the middle of the biomass and sagittal views (rectangle) are shown. Magnification used was 200X. Scale bars represent 50 µm.

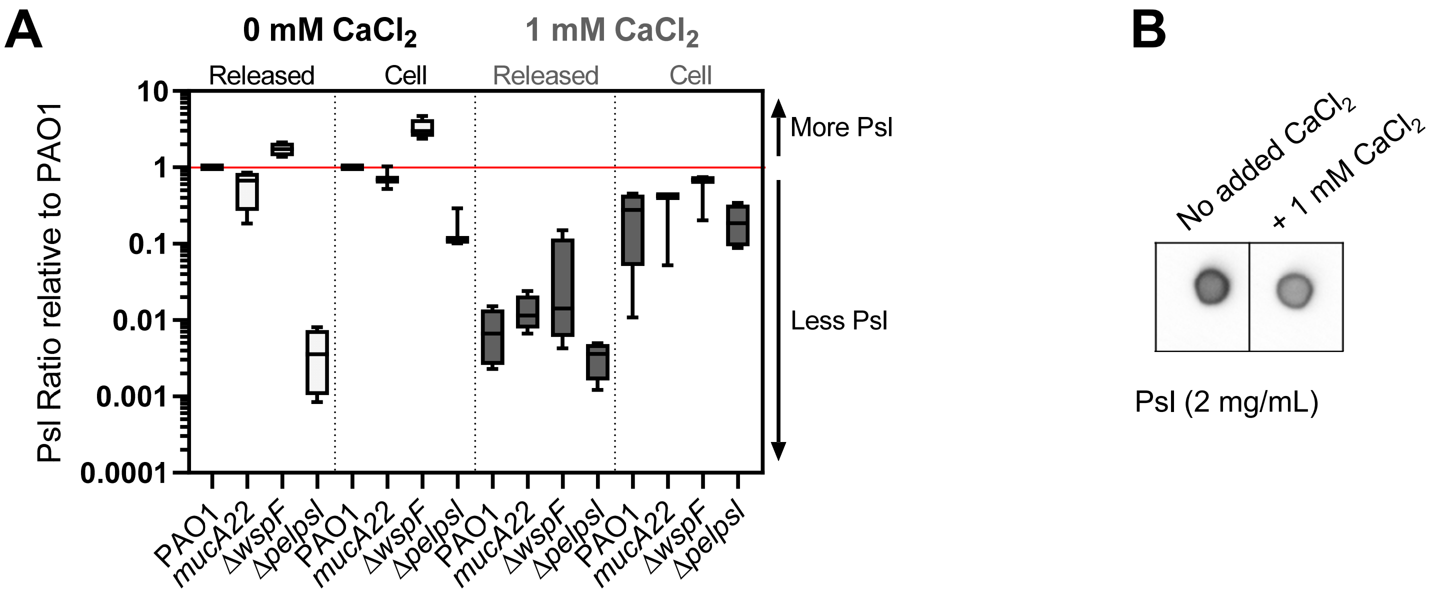

Supplemental Figure 4. Effect of $CaCl_2$ on released and cell-associated Psl. (A) Relative to PAO1 grown in EPRI medium with 0 mM $CaCl_2$ (white boxes), Psl is absent in all tested strains when 1 mM $CaCl_2$ (grey boxes) is added to the growth medium. α-Psl immunoblots (representative blot shown in Fig. 4C) from 4 independent experiments were quantified by measuring mean intensity of each sample dot in FIJI. Within each experiment, measures were adjusted to the intensity of a blank portion of the blot, then divided by the intensity of PAO1 released or cell-associated ("cell") Psl from the 0 mM $CaCl_2$ condition. The boxes extend from the 25th to 75th percentiles, the whiskers represent the minimum and maximum values and the line in the middle of the box is plotted at the median value. (B) 1 mM $CaCl_2$ does not interfere with the ability of the α-Psl antibody to recognize Psl. An α-Psl immunoblot was performed on solutions of purified Psl (2 mg/ml) prepared in EPRI medium (with or without 1 mM $CaCl_2$ added immediately prior to blotting solutions onto the membrane).

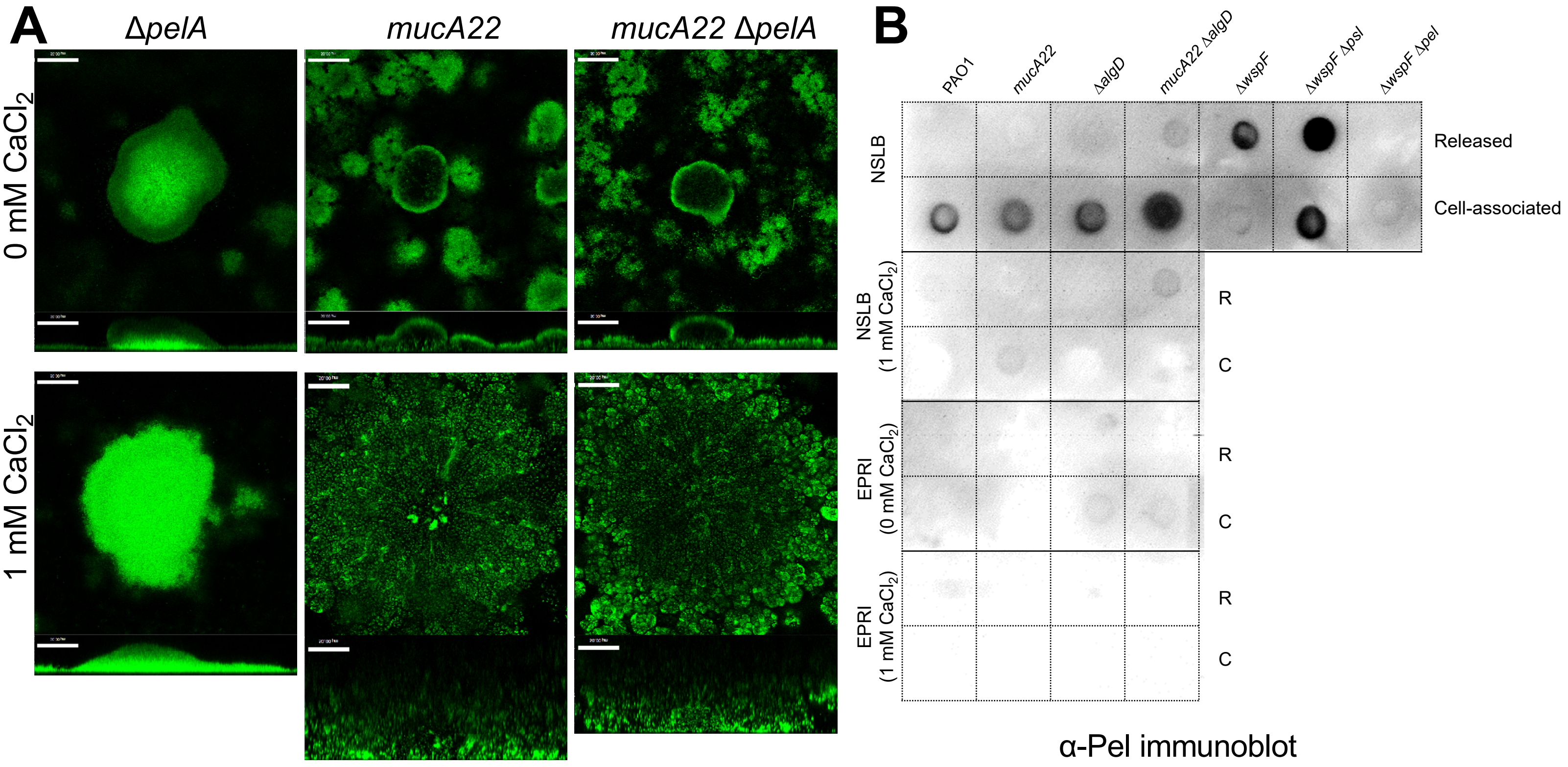

Supplemental Figure 5. Pel is not required for the formation of calcium cross-linked mucoid biofilms. (A) Biofilm growth of PAO1 strains deficient in Pel production (Δ*pelA*). Representative confocal images are from biofilms cultivated for 72 h under continuous flow conditions. Cells are expressing GFP (pseudo-colored green) under control of a constitutively active promoter. Horizontal cross-sections (square) from the middle of the biomass and sagittal views (rectangle) are shown. Magnification used was 200X. Scale bars represent 50 μm. (B) Anti-Pel immunoblot performed on released (R) and cell-associated (C) fractions of statically-grown biofilms. Cultures were grown for 24 h in wells of a 6-well tissue culture plate at 37°C in 5 mL of EPRI medium or NSLB, supplemented with $CaCl_2$ (1 mM) as noted.

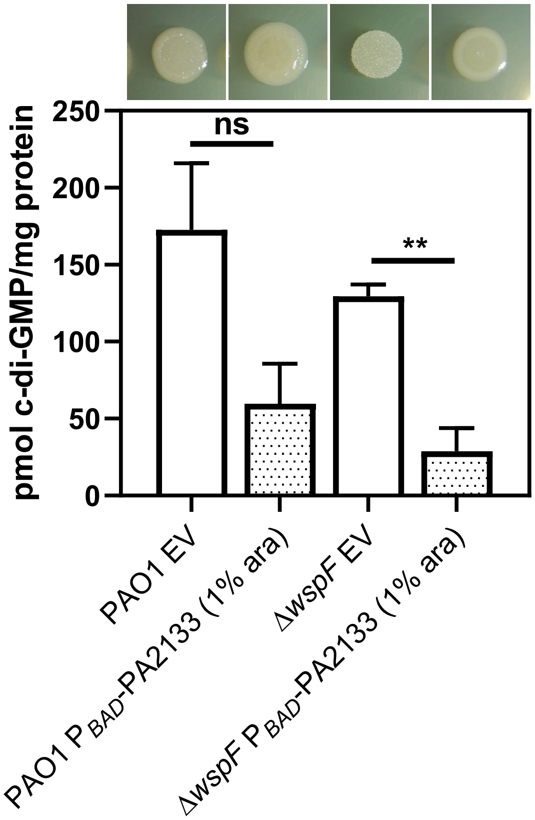

Supplemental Figure 6. Overexpression of phosphodiesterase PA2133 decreases c-di-GMP and eliminates rugosity in PAO1 Δ*wspF*. c-di-GMP was quantified from cultures statically-grown in EPRI medium for 24 h at 37°C. White bars: without arabinose; dotted bars: with 1% arabinose. Representative colonies grown on NSLB agar for 36 h at 37°C are shown above corresponding bars. Empty vector (EV) is pJN105. Unpaired, two-tailed t-test; **, P<0.01; ns, not significant, P>0.05.

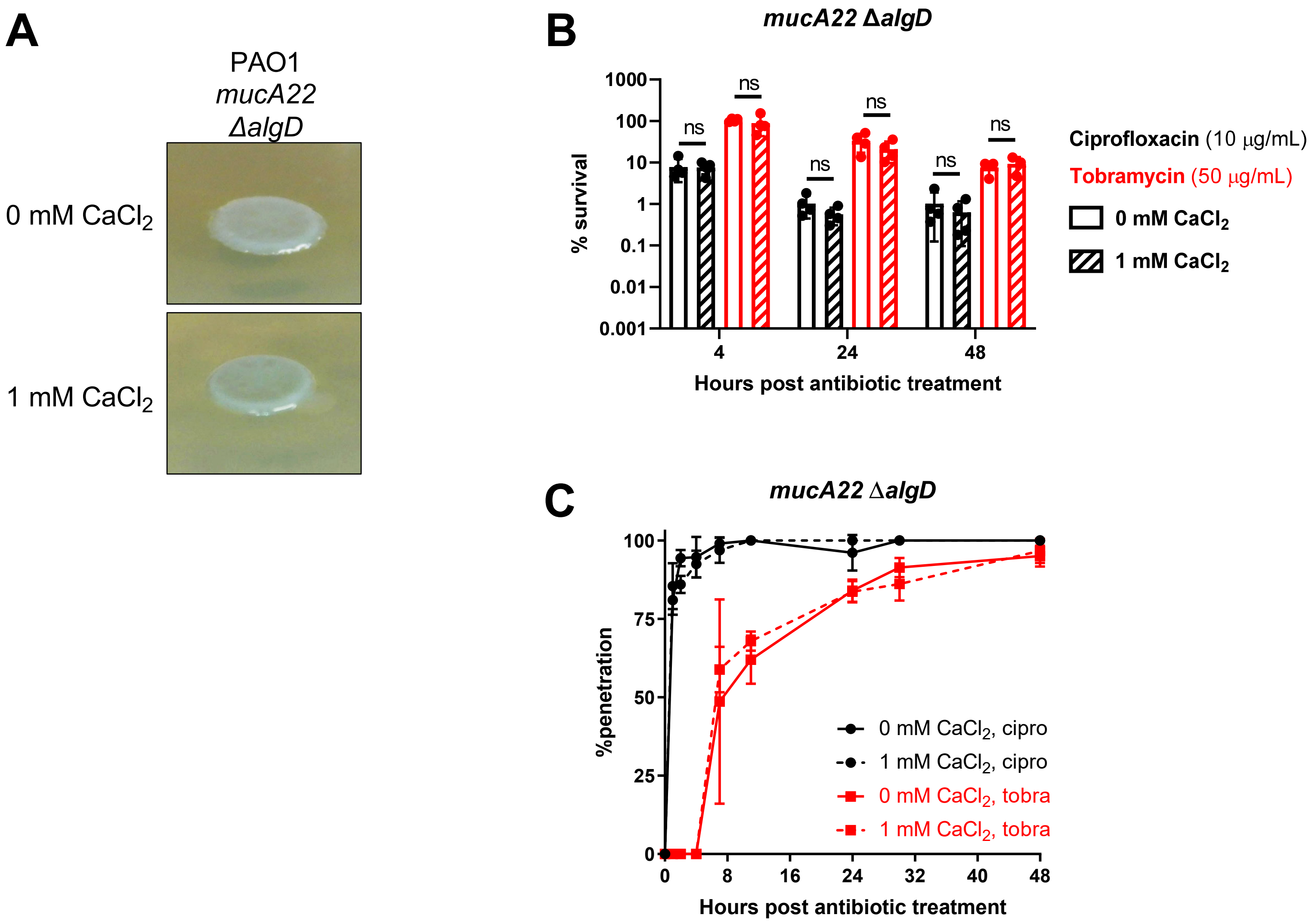

Supplemental Figure 7. Penetration of tobramycin and ciprofloxacin through non-mucoid biofilms. (A) Morphology of representative colony biofilms of PAO1 *mucA22* used in antibiotic penetration experiments. Overnight planktonic cultures grown in NSLB were diluted (OD_{600} = 0.1) and spotted (5 µL) onto UV sterilized membranes placed on the agar surface. Liquid was allowed to absorb into the agar before growing inverted for 24 h at 37°C. (B) Tobramycin (red) and ciprofloxacin (black) killing of PAO1 *mucA22* Δ*algD* biofilms. Colony biofilms were resuspended in 10 mL PBS, pH 7.4 at noted time points after placement on antibiotic-containing agar, and serially diluted and plated on NSLB agar. Colony forming units (CFU) were counted after 24 h of growth at 37°C and percent survival was calculated using CFU counts from time = 0 h (pre-antibiotic exposure). n = 4. Unpaired, two-tailed t-test; ns, not significant, P>0.05. (C) Percent penetration of antibiotic through colony biofilms grown on agar supplemented with calcium and antibiotic. Colonies were spotted onto a 0.22 µm membrane placed on agar with or without calcium and grown at 37°C for 24 hr. Another membrane and a wetted paper disk were placed on top of the colony then the whole apparatus was transferred to plates containing antibiotics and were incubated at 37°C for up to 48 h. Antibiotic penetration was measured by placing top paper disks onto a lawn of antibiotic-sensitive *E. coli* and measuring zones of inhibition after 8 h of growth at 37°C. Percent penetration was calculated by dividing the diameter of zone produced by a disk from on top of a colony biofilm by the zone produced by a disk placed on top of just two membranes (a "sterile" apparatus) from the same time point, multiplied by 100%. Ciprofloxacin is shown in black, tobramycin in red. Dashed lines indicate the presence of 1 mM $CaCl_2$ in the agar throughout the experiment. n = 4. Unpaired two-tailed t-tests comparing 0 to 1 mM $CaCl_2$ in tobramycin and ciprofloxacin treatments, respectively, at each time point were not significant (ns, P>0.05).

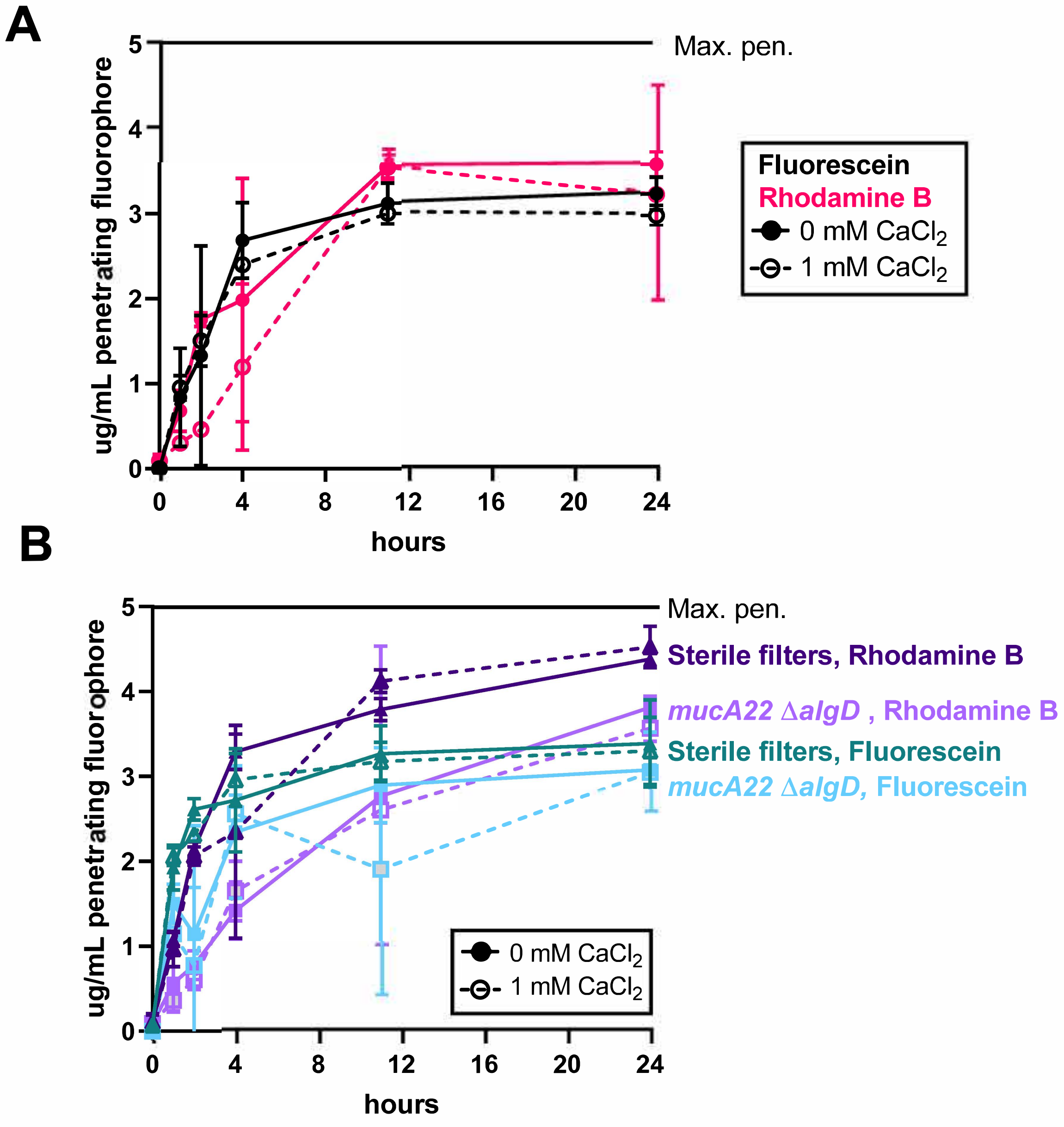

Supplemental Figure 8. Molecule charge does not affect penetration through colony biofilms. Penetration of fluorescein (negatively-charged) and rhodamine B (positively-charged) through mucoid colony biofilms (A) or non-mucoid and sterile filters (B) on agar supplemented with calcium and fluorophore. Colonies were spotted onto a 0.22 µm membrane placed on agar with or without calcium and grown at 37°C for 24 hr. Another membrane and a wetted paper disk were placed on top of the colony then the whole apparatus was transferred to plates containing fluorophores (50 µg/mL) and were incubated at 37°C for up to 24 h. Fluorophore penetration was measured by eluting accumulated fluorophore in the paper disks into 225 µL PBS, pH 7.4 and measuring fluorescence. Concentration of fluorophore was determined using standard curves generated from known concentrations of fluorophores. In (A) rhodamine B is shown in pink and fluorescein is shown in black. In (B) rhodamine B is shown in violet and fluorescein is shown in teal. Solid lines indicate the absence of supplemental calcium and dashed lines indicate the presence of 1 mM $CaCl_2$ in the agar throughout the experiment. n = 3.

References

1. Reichhardt C, Wong C, Passos da Silva D, Wozniak DJ, Parsek MR. 2018. CdrA Interactions within the *Pseudomonas aeruginosa* Biofilm Matrix Safeguard It from Proteolysis and Promote Cellular Packing. MBio 9:e01376-18.
2. Zhao K, Tseng BS, Beckerman B, Jin F, Gibiansky ML, Harrison JJ, Luijten E, Parsek MR, Wong GCL. 2013. Psl trails guide exploration and microcolony formation in *Pseudomonas aeruginosa* biofilms. Nature 497:388–391.
3. Choi KH, Schweizer HP. 2006. mini-Tn7 insertion in bacteria with single *att*Tn7 sites: Example *Pseudomonas aeruginosa*. Nat Protoc 1:153–161.
4. Borlee BR, Goldman AD, Murakami K, Samudrala R, Wozniak DJ, Parsek MR. 2010. *Pseudomonas aeruginosa* uses a cyclic-di-GMP-regulated adhesin to reinforce the biofilm extracellular matrix. Mol Microbiol 75:827–842.
5. Holloway BW. 1955. Genetic recombination in *Pseudomonas aeruginosa*. J Gen Microbiol 13:572–581.
6. Fyfe JA, Govan JR. 1980. Alginate synthesis in mucoid *Pseudomonas aeruginosa*: a chromosomal locus involved in control. J Gen Microbiol 119:443–450.
7. Hentzer M, Teitzel GM, Balzer GJ, Heydorn A, Molin S, Givskov M, Parsek MR. 2001. Alginate Overproduction Affects *Pseudomonas aeruginosa* Biofilm Structure and Function. J Bacteriol 183:5395–5401.
8. Hickman JW, Tifrea DF, Harwood CS, Greenberg EP. 2005. A chemosensory system that regulates biofilm formation through modulation of cyclic diguanylate levels. Proc Natl Acad Sci U S A 102:14422–14427.
9. Irie Y, Starkey M, Edwards AN, Wozniak DJ, Romeo T, Parsek MR. 2010. *Pseudomonas aeruginosa* biofilm matrix polysaccharide Psl is regulated transcriptionally by RpoS and post-transcriptionally by RsmA. Mol Microbiol 78:158–72.
